# The Effects of Broadband Elicitor Duration on Transient-Evoked Otoacoustic Emissions and a Psychoacoustic Measure of Gain Reduction

**DOI:** 10.1007/s10162-025-01011-1

**Published:** 2025-11-25

**Authors:** William B. Salloom, Hari Bharadwaj, Elizabeth A. Strickland

**Affiliations:** 1 Department of Speech, Language, and Hearing Sciences, Purdue University, West Lafayette, IN 47907 USA; 2 Weldon School of Biomedical Engineering, Purdue University, West Lafayette, IN 47907 USA

**Keywords:** Psychoacoustics, Otoacoustic emissions, Medial olivocochlear reflex, Auditory masking, Cochlea, Gain reduction

## Abstract

**Purpose:**

Measures of the human medial olivocochlear reflex (MOCR) typically rely on long duration (> 100 ms) or continuously presented broadband elicitors. MOCR gain reduction measured by otoacoustic emissions (OAE) exhibits multiple time constants, including in the hundreds of milliseconds, when elicited by broadband noise. Psychoacoustic studies of gain reduction have largely adopted these elicitor characteristics, but less is known about how broadband elicitor duration affects auditory perception. Additionally, the literature on the relationship between psychoacoustic and OAE measures of gain reduction has yielded mixed results, which is counterintuitive if both measures reflect the same mechanism. In this study, the effects of ipsilateral broadband elicitor duration were evaluated using forward masking psychoacoustic and transient-evoked OAE (TEOAE) paradigms in individuals with normal hearing (N = 19; m = 7, f = 12).

**Methods:**

Ipsilateral pink broadband noise was used as the elicitor in both experiments, presented at 50 dB SPL (50–800 ms) for the psychoacoustic measures and 50 dB FPL (50–400 ms) for the TEOAE measures. Gain reduction was quantified as the change in signal threshold (2 kHz) and the change in TEOAE level (1/3rd-octave band centered at 2 kHz) with and without the presence of the elicitor.

**Results:**

The average time constants for psychoacoustic and TEOAE gain reduction were similarly short (< 100 ms), with near-maximal effects observed for elicitor durations of 200 ms. However, individual comparisons of TEOAE and psychoacoustic gain reduction revealed mixed results. Potential factors contributing to this discrepancy are discussed.

**Conclusion:**

The human MOCR reduces cochlear gain on relatively short time scales (< 100 ms) with ipsilateral broadband elicitors.

**Supplementary Information:**

The online version contains supplementary material available at 10.1007/s10162-025-01011-1.

## Introduction

A defining feature of the human auditory system is its ability to operate across an exceptionally wide dynamic range of approximately 100 dB, spanning from the faintest audible sounds at absolute threshold to the threshold of tolerable loudness. To support this wide perceptual dynamic range across diverse listening environments, the auditory system likely employs multiple mechanisms for optimizing sound encoding [1]. One such mechanism involves adapting the dynamic range of the afferent neural pathway based on the listening environment and incoming sound characteristics [2–4]. The medial olivocochlear reflex (MOCR) is a neural circuit that may play a significant role in this adaptation. The MOCR is a bilateral, sound-activated feedback loop with efferent fibers originating in the superior olivary complex of the brainstem and projecting to each cochlea. These efferent fibers synapse at the base of the outer hair cells (OHCs) [5]. MOCR activation reduces OHC gain in a frequency-specific manner relative to the elicitor sound, leading to decreased basilar membrane (BM) movement [6]. This gain reduction is thought to adjust the dynamic range, as neurophysiological evidence in animals shows that MOCR activation enhances auditory nerve responses to transient sounds in the presence of background noise, and shifts the rate-level functions to higher input sound levels in quiet [7, 8]. Perceptually, the MOCR may enhance the fluctuation profile of complex sounds [9], increase sensitivity to intensity changes in auditory perception [10, 11], and improve speech intelligibility in noise [12].

The temporal dynamics of MOCR gain reduction (referred to as gain reduction through the rest of the paper), including activation and decay, have long been an area of research interest. Given that cochlear gain reduction may enhance speech-in-noise perception [12], understanding the speed of gain adjustment and identifying the time periods most beneficial for perception could provide valuable insights. These topics have become increasingly relevant in translational research, as hearing aid [13] and cochlear implant [14] algorithms are being developed to incorporate MOCR-inspired gain reduction to improve speech-in-noise performance. It is likely that individuals using these devices and individuals who have significant peripheral hearing loss do not benefit from MOCR gain adjustment [15], potentially contributing to their poorer speech-in-noise intelligibility compared to normal-hearing listeners [16]. Modeling studies support this concept, showing improved speech intelligibility in noise when efferent-inspired gain reduction enhances the contrast between speech signals and background noise [17, 18]. The temporal properties of MOCR gain reduction are also critical for physiological models of the auditory system. These properties have been incorporated into auditory nerve models [19, 20] and subcortical models [21]. Understanding the MOCR time course can guide the design of appropriate stimuli for physiological, psychoacoustic, and modeling experiments.

The temporal dynamics of the MOCR in humans have been studied most extensively using otoacoustic emissions (OAEs). OAEs are low intensity sounds generated by OHC amplification that can be recorded noninvasively from the ear canal [5]. The magnitude of the MOCR effect is typically assessed by comparing otoacoustic responses to a probe stimulus with and without elicitor sounds that activate the MOCR [22–26]. Often the elicitors have been presented to the contralateral ear to avoid non-MOCR effects. Broadband noise elicitors are often used because they are particularly effective in activating gain reduction [27, 28]. MOCR activation with these stimuli configurations generally results in a reduction of OAE magnitude. Backus and Guinan (2006) investigated the time course of the MOCR by measuring changes in stimulus-frequency OAEs (SFOAEs) in response to contralateral broadband noise elicitors. They identified three time constants characterizing MOCR gain reduction: a fast component (~ 60–80 ms), a medium component (~ 290–350 ms), and a slow component (~ 11–39 s). Additionally, there is a delay of approximately 20–25 ms for both the onset and offset of the MOCR-induced gain reduction, with the response gradually building up after the elicitor onset and decaying after the elicitor offset [23, 24]. This sluggish onset and offset response reflects the time required for the MOCR to fully activate and then gradually diminish. These time constants, from tens of milliseconds to tens of seconds, align with data from other OAE-based MOCR paradigms in humans (DPOAEs: [23, 29, 30]; TEOAEs: [31]). MOCR inhibitory effects operating on both fast (tens of milliseconds) and slow (tens of seconds) time scales have been observed in mammals by measuring sound-evoked responses at the level of the basilar membrane (BM) [32] and auditory nerve [33] while activating the MOCR through electrical stimulation of the olivocochlear bundle (OCB).

Psychoacoustic techniques have also been used to measure gain reduction, potentially mediated by the MOCR. These methods have been discussed in detail in Salloom et al. (2023) [34], but briefly, in a phenomenon called “overshoot” [35] or the “temporal effect” [36], a tone may be detected at a lower signal-to-noise (SNR) ratio within a simultaneous masker when delayed from the onset of a masker or there is preceding sound before the signal and masker. The time course of the temporal effect has been measured, and it plateaus around 200 ms after masker or preceding sound onset [35]. One study comparing OAE and behavioral measures of overshoot found comparable short time constants for both measures (64.8 ms behavioral, 72 ms OAE; [37]), suggesting that MOCR-mediated gain reduction may predominantly operate on a short time scale in behavior. However, with simultaneous masking, excitatory masking and suppression may also contribute to observed effects, both of which could be influenced by MOCR-induced gain reduction.

Psychoacoustic forward masking paradigms have been used to study gain reduction. Forward masking avoids confounds such as two-tone suppression. These paradigms have typically used short duration tonal signal and masker stimuli to measure effects consistent with cochlear processing. As mentioned earlier, OAE studies have found that there is a 20–25 ms delay between the onset of the elicitor and the onset of the MOCR buildup. If the duration of the signal and masker are shorter than the onset of the MOCR buildup, then gain reduction by the MOCR should not occur. These paradigms use maskers at the signal frequency (on-frequency) and maskers approximately an octave below the signal frequency (off-frequency). The growth of masking produced by the off-frequency masker is hypothesized to provide an estimate of the cochlear input–output (IO) function [38]. Then, an elicitor is added before the signal and masker, and the IO functions are re-measured. The difference in threshold (either signal or masker threshold) with and without the elicitor shifts the linear, low-level portion of the IO function to higher signal levels, which is consistent with gain reduction [15, 39–42]. The differential processing of on- and off-frequency masking at the signal frequency place with the presence of an elicitor serves as the basis to study gain reduction and is further detailed in Supplementary File 1; Supplementary Fig. 1A-B. It has also been explained in detail in our prior work [34].

Many psychoacoustic studies measuring gain reduction, or measures thought to be indicative of MOCR activation, have adopted the use of very long duration (> 100 ms) or continuously presented broadband elicitors (e.g., [42–47]). This is largely because it is assumed that the best way to elicit gain reduction in psychoacoustic paradigms is to use elicitor characteristics based on OAE paradigms to maximize its effect. However, prior to the current study, the effects of the duration of broadband elicitors on similar psychoacoustic tasks have largely been unexplored. Additionally, the relationship between gain reduction measured psychoacoustically and using OAEs as a function of elicitor duration are unknown. A few of our recent studies shed light onto these unknowns. Salloom et al. (2023) investigated the time course of gain reduction using a psychoacoustic forward masking paradigm with ipsilateral broadband noise elicitors spanning durations from 50 to 800 ms. Gain reduction was measured under two conditions: with a short tonal masker present (masker present) and with a short delay between the elicitor offset and signal onset (masker absent). They reported time constants of 46 ± 5 ms for the masker-present condition and 78 ± 13 ms for the masker-absent condition. For both conditions, threshold shifts were maximal or near-maximal within ~ 200 ms of elicitor activation [34], consistent with the fast time constant of MOCR activation [24]. In a follow-up study, Salloom et al. (2024) measured gain reduction time courses using both psychoacoustic forward masking and transient-evoked OAE (TEOAE) paradigms, as a function of ipsilateral broadband elicitor duration. The elicitor durations ranged from 50 to 800 ms in the psychoacoustic paradigm and 50 to 400 ms in the TEOAE paradigm. Changes in the TEOAE were analyzed in terms of magnitude and phase. When both magnitude and phase were accounted for, the estimated time constants were relatively short (~ 53 ms) and closely matched the psychoacoustic time constants (~ 62–63 ms). However, when only TEOAE magnitude was considered, the time constants were longer (~ 97 ms). Across all conditions, MOCR effects reached maximal or near-maximal levels within ~ 200 ms of elicitor activation. These findings support the idea of a shared gain reduction mechanism, likely mediated by the MOCR, as both psychoacoustic and TEOAE time constants align with the fast MOCR buildup [24]. Importantly, these results suggest that gain reduction on these timescales is significantly shorter than what is observed with longer-duration (> 100 ms) or continuously presented elicitors often used in OAE paradigms [48]. However, the Salloom et al. (2024) study did not include direct comparisons between the TEOAE and psychoacoustic measures of gain reduction or time constants for individual subjects.

To our knowledge, no prior reports have directly studied the relationship of elicitor duration effects on psychoacoustic and OAE-based measures of gain reduction. If psychoacoustic measures of gain reduction and MOCR strength, as measured via OAEs, reflect the same underlying mechanism, it might seem intuitive to expect a positive correlation between the two. However, results from the literature reveal that the relationship between various psychoacoustic measures of gain reduction or related tasks (i.e., intensity discrimination, psychoacoustic tuning curve (PTC), signal-in-noise detection, overshoot) and OAE-based measures of gain reduction yield mixed or uncorrelated results [37, 43–46, 49, 50]. Multiple potential factors may account for this lack of a consistent relationship (see [11, 46] for review). Some less frequently discussed factors for these discrepancies are addressed here. First, methodologies across prior studies often vary drastically between psychoacoustic and OAE measurements. In some cases, it is unclear whether the paradigms are truly comparable, as different response mechanisms may be assumed to underlie each measure. Second, several studies have tested relatively small sample sizes (< 12 individuals), meaning that variability across subjects could obscure relationships between measures and substantially weaken statistical power if a relationship does exist. Third, some studies have employed contralateral noise elicitors to activate the MOCR, even though psychoacoustic evidence suggests that ipsilateral elicitors produce larger gain reduction effects at equivalent sound pressure levels [51]. Thus, contralateral elicitor paradigms may underestimate the strength of gain reduction compared to ipsilateral measurements.

In the current study, we further investigated the temporal dynamics of psychoacoustic and physiological measures of gain reduction using ipsilateral broadband elicitors in a forward masking paradigm. Building on the work of Salloom et al. (2024), we incorporated additional analyses and directly compared both individual and averaged time constants for gain reduction measured across psychoacoustic and OAE paradigms. We also compared the magnitudes of gain reduction (i.e., TEOAE level changes and psychoacoustic threshold shifts) as a function of elicitor duration. These comparisons were intended to determine whether physiological responses can predict psychoacoustic responses, thereby supporting the hypothesis that a shared underlying mechanism, gain reduction mediated by the MOCR, accounts for both. To address the potential factors outlined above regarding previous comparisons of psychoacoustic and OAE measures of gain reduction, the current study implemented several improvements. We used ipsilateral broadband elicitors to elicit a stronger effect than contralateral elicitors. A larger subject pool (N = 19) was tested to increase statistical power and to make individual comparisons. Finally, we used similar stimuli and parallel paradigms across our psychoacoustic and physiological measurements to ensure a fair comparison. Overall, the results of this study highlight how the peripheral auditory system adapts to broadband noise and how these processes, along with their timescales, influence auditory perception.

## Experiment 1: Psychoacoustic Gain Reduction as a Function of Broadband Elicitor Duration

### Methods

#### Subjects

##### Audiological Testing

Nineteen subjects (7 male and 12 female) completed the psychoacoustic and otoacoustic experiments in the current study. Their ages ranged from 19 to 35 years (median = 24 years) at the time of testing. All subjects had normal auditory function, determined through a battery of audiologic measures. All subjects had clinically normal pure tone thresholds [≤ 15 dB hearing level (HL) at audiometric frequencies between 250 and 8000 Hz]. Distortion product otoacoustic emissions (DPOAEs) were present (Bio-logic system, Natus Medical, Inc., Pleasanton, CA) from 1500 to 8500 Hz (minimum criteria of –6 dB SPL distortion product and 6 dB SNR for 10 of 12 frequencies tested with no consecutive absent responses). Tympanograms (Tympstar, Grason-Stadler, Inc.) were normal (type A), indicating normal middle-ear function. Ipsilateral middle-ear muscle reflex (MEMR) thresholds were measured using white broadband noise elicitors and 226-Hz probe tones. The clinical MEMR thresholds were measured with respect to the probe ear for the experiments (right ear for all listeners except for S18). The clinical MEMR thresholds were measured in dB HL and converted to corresponding dB SPL units for fair comparison to the experimental elicitors used in the current study. To do so, noise levels from the immittance equipment were recorded from a sound level meter attached to a Zwislocki coupler mounted in a KEMAR ear. The noise levels in dB SPL were approximately 8 dB higher than the nominal levels in dB HL. Overall, no subject’s MEMR threshold to broadband noise was below 68 dB SPL, a level that is well above the elicitor level used in the experiments (50 dB SPL and 50 dB FPL). Furthermore, a wideband acoustic immittance (WAI) procedure was conducted to estimate each subject’s ipsilateral MEMR threshold to ensure that the stimuli used in the current study did not evoke the MEMR. WAI measures are considered a more sensitive measure of MEMR thresholds compared to typical clinical tympanometry using a single probe tone [52]. The WAI procedure and stimuli used closely followed those used in Bharadwaj et al. (2022) [53], and have been used in our prior work [34]. The results from the WAI MEMR measures were consistent with the clinical MEMR thresholds, with no subject’s WAI MEMR threshold below 50 dB forward pressure level (FPL). In conclusion, our rigorous assessments confirm that the gain reduction experiments in this study are not confounded by MEMR effects.

Two additional potential subjects started the study but did not complete it. One of these subjects had tinnitus, which would have interfered with our tone detection tasks and confounded the results. The other potential subject could not consistently perform the behavioral tasks and was discontinued from the study after multiple days of unsuccessful practice sessions. Data from these subjects are not reported here. All subjects were paid for their time in the study except for S1, who is the first author. Other subjects were recruited via fliers on the Purdue campus. All research was conducted under a research protocol approved by the Institutional Review Board at Purdue University to safeguard the rights, safety, and well-being of our subjects.

#### Psychoacoustic Measures of Gain Reduction

##### Stimuli

**Fig. 1:**
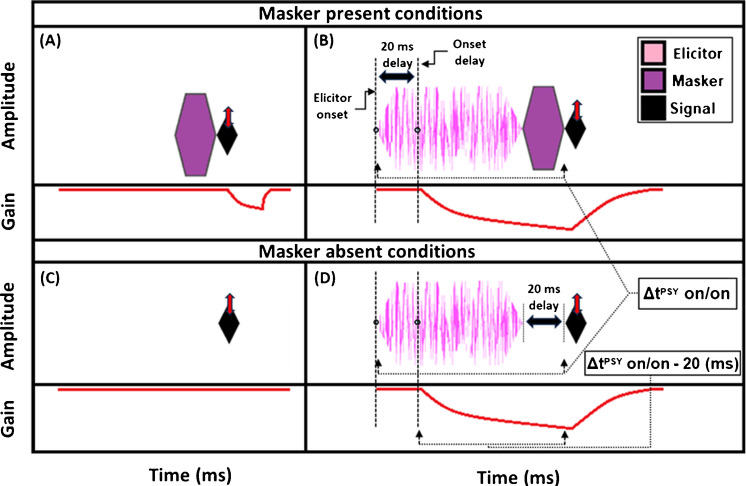
Schematics of the stimuli used for the masker present method (**A** and **B**) and the masker absent method (**C** and **D**), respectively. Accompanying the stimuli are estimations of the time course of gain reduction (bottom portion in each panel). In the masker present condition, the masker depicted represents either on- or off-frequency conditions. Both on- and off-frequency maskers had a duration of 20 ms, which matches the delay between the elicitor offset and signal onset in the masker absent condition. Elicitor durations ranged from 50 to 800 ms. The double-headed arrow (red) indicates that the signal was adaptively varied, while the masker was fixed at a level that shifted the signal by 5 dB with no elicitor present. To account for the onset delay (bottom), the total time of elicitation starts 20 ms after elicitor onset and ends at the onset of the signal [Δt^PSY^ on/on - 20 (ms)]. Because the onset delay of the MOCR and the duration of the masker/gap are equal, ‘t’ is equal to the elicitor duration for each data point in the psychoacoustic experiment

Estimates of gain reduction were made at 2 kHz using two forward masking techniques that rely on the timing of cochlear gain reduction via the MOCR. The 2-kHz signal frequency was chosen because it has shown gain reduction effects in both human psychoacoustic [45, 51, 54] and physiological studies (OAEs: [55, 56]). The technique used to measure gain reduction with the use of short duration maskers (“masker present” conditions) will be explained first (Fig. 1, top panels). The signals used in the behavioral experiments were 10-ms sinusoids, including 5-ms cos^2^ onset and offset ramps. This duration was used to ensure that spectral spread was within one auditory filter bandwidth [54]. The measurement process began by determining the threshold for the signal in quiet. Next, the masker levels needed to mask a signal fixed at 5 dB sensation level (SL) were determined. A 5-dB shift in signal threshold was desired so that the signal was fixed on the lower leg (the linear low-input sound level portion) of the cochlear IO function. Gain reduction is largest in this region of the IO function (psychoacoustically: [39, 41]; physiologically: [32]). The masker duration was 20 ms (including 5-ms cos^2^ ramps). This duration was intentionally kept short to avoid eliciting the MOCR during the signal presentation, as previous studies have indicated a 20 to 25-ms delay between elicitor onset and MOCR onset [23, 24]. The elicitor was a 50-dB SPL pink broadband noise (0.25–10 kHz), and elicitor durations ranged from 50 to 800 ms (including 5-ms cos^2^ onset and offset ramps). These elicitor durations were used in recent studies investigating the time course of gain reduction with broadband elicitors [34, 48], and should overlap with the entirety of the buildup of the MOCR [23, 24]. Pink noise has a spectrum level that decreases by 3 dB per octave. This elicitor provides a more accurate comparison of gain reduction across frequency, as pink noise will excite auditory filters across the frequency range with approximately equal energy. Additionally, previous studies have found broadband noise stimuli to be particularly effective elicitors of cochlear gain reduction [27, 28, 44]. The pink noise was presented ipsilaterally with respect to the masker and signal. Ipsilateral elicitors produce significantly larger cochlear gain reduction compared to contralateral elicitors of the same sound pressure level in psychoacoustical experiments [51]. 50 dB SPL is a relatively low elicitor level compared to many other studies in humans measuring MOCR effects, which typically use 60-dB SPL elicitors. A previous study conducted in our lab [51] found that a 50-dB SPL elicitor was nearly as effective as a 60-dB SPL elicitor at 2 kHz and is less likely to elicit the MEMR.

For gain reduction measurements, we used both on-frequency (2 kHz) and off-frequency (0.6 times the signal frequency, 1.2 kHz) sinusoidal maskers. The signal level was set at 5 dB SL, and the masker level was adjusted to find the level needed to just mask the signal. By measuring both on- and off-frequency masked threshold, we were able to test whether the effects of the elicitor on signal threshold were consistent with cochlear gain reduction (explained below). To verify that the maskers were equally effective at masking the signal, the on- and off-frequency maskers were fixed at the thresholds determined earlier, and the signal was varied to check that the signal thresholds were raised to 5 dB SL. On- and off-frequency masked signal thresholds were considered similar if the difference between the two conditions was less than 3 dB. If signal threshold with the fixed maskers differed by more than this amount, the masker level was adjusted, and the signal threshold was remeasured until signal thresholds were within 3 dB of one another.

A reduction in gain by the elicitor is predicted to produce a larger shift in signal threshold when an off-frequency masker is used compared to an on-frequency masker [42, 43, 51, 54]. This is because the off-frequency masker is processed linearly at the signal frequency place and thus is not affected by gain reduction, whereas the signal and the on-frequency masker are nearly equally affected by gain reduction (see Supplementary File 1; Supplementary Fig. 1). This contrasts with the prediction of temporal integration of the elicitor and masker, also called additivity of masking [57]. With temporal integration, it would be expected that the on- and off-frequency conditions should produce equal shifts in thresholds with the addition of the elicitor. However, in this study, we propose that masking by the masker may occur within a temporal window (because the duration is too short for gain reduction to affect the signal) while masking from the elicitor occurs by gain reduction. For our analyses, we focused on the shift in signal thresholds with an off-frequency masker following the presence of an elicitor. These shifts have been interpreted as indicative of a change in gain. Therefore, in the current study, the difference in signal threshold between the off-frequency masked signal with (Fig. 1B) and without (Fig. 1A) the elicitor will be referred to as the “masker present” gain reduction estimate. Note that the schematic masker in the masker present condition in Fig. 1 represents either the on- or off-frequency masked condition. The measurement of on- and off-frequency masked signal thresholds is analogous to the tip- and tail-response of a PTC measured at the signal frequency place.

A second method was also used to estimate gain reduction. Instead of using an off-frequency masker to fix the signal on the lower leg of the IO function, the baseline condition was the quiet threshold of the signal (Fig. 1C), which was then compared to the signal threshold with an elicitor and a 20-ms delay between the elicitor offset and signal onset (Fig. 1D). The difference between these two conditions will be referred to as the “masker absent” estimate of gain reduction. Previous studies have found that signal threshold shifts by the elicitor for the off-frequency masked and masker absent conditions are similar between the two methods [34, 51, 54]. We used this masker absent condition to determine if it exhibited a similar growth pattern to the off-frequency masked condition and because it was more similar to the paradigm used by Oxenham and Plack (2000) which studied forward masking as a function of masker duration [58]. Furthermore, the masker absent condition was designed to be as similar as possible to the TEOAE stimuli used in the current study (see Fig. 5). In the current study, the growth in signal threshold with elicitor duration was measured for the masker present and absent conditions. From these thresholds, gain reduction was measured in terms of magnitude and time constants. As mentioned in the current section, there was always 20 ms between the offset of the elicitor and the onset of the signal, whether a masker was present (Fig. 1B) or absent (Fig. 1D). This is estimated to be equal to the offset delay of gain reduction from the elicitor. We accounted for this delay in our gain reduction measures to include the total duration from the onset of the elicitor to the onset of the signal (Δt^PSY^ on/on) minus the onset delay. The onset delay was estimated to be 20 ms (Figs. 1B, 1D) [23, 24]). This means the data points used in our time constant estimations were equal to the elicitor durations used in the psychoacoustic experiment [depicted in Fig. 1 (bottom panel)]. Therefore, each data point in the magnitude and time constant estimations corresponded to the duration of the elicitor [50, 65, 100, 200, 400, and 800 ms] when the correction for onset delay of the MOCR and the duration of the masker/gap were accounted for (Δt^PSY^ on/on - 20 ms).

The psychoacoustic conditions in this study follow some underlying assumptions that are addressed here. First, it is assumed that the listener makes use of an auditory filter with a center frequency at or close to the signal frequency. Second, only the components of the masker passing through this filter have any effect in masking the signal. Third, signal threshold is assumed to correspond to a certain signal-to-masker ratio at the output of the auditory filter. These assumptions are based on the power spectrum model of forward masking [59, 60], and are shown schematically in Supplementary Fig. 1.

##### Procedure

All psychoacoustic measures were conducted in a double-walled sound-attenuating booth. Stimuli were generated with custom MATLAB software [61] with a Lynx TWO-B sound card (Lynx Studio Technology, Inc., Costa Mesa, CA). The stimuli were then passed through a headphone buffer (TDT HB6, Tucker-Davis Technologies, Alachua, FL) and delivered to one ear through an Etymotic ER-2 (Etymotic Research, Inc., Elk Grove Village, IL) insert earphone. All subjects had insert earphones in both ears. The insert earphones have a flat frequency response at the eardrum from 250 to 8000 Hz. High pass noise (from 1.2 times the signal frequency to 10 kHz) was used to reduce the possibility of off-frequency listening [62] for all parts of the experiment except during quiet threshold measurements. The high pass noise began 50 ms before the onset of the stimuli and ended 50 ms after the signal offset and was 50 dB spectrum level below the signal level.

All psychoacoustic measurements utilized a three-interval forced-choice (3IFC) task using a MATLAB GUI, in which only one of the choices contained the signal. Each interval was visually marked on the computer screen, and intervals were separated by 500 ms of silence. Subjects could use either a mouse or the keyboard to indicate which interval contained the signal. Visual feedback was given for correct and incorrect responses. Signal and masker levels were adjusted to estimate a response threshold of 70.7% correct [63]. For signal threshold measures (quiet thresholds and measures of gain reduction), if the subject chose correctly over two consecutive trials, the level of the signal decreased, while an incorrect response would cause the level of the signal to increase (two down, one up). For masking thresholds, if the subject chose correctly over two consecutive trials, the level of the masker increased, while an incorrect response would cause the level of the masker to decrease (two up, one down). The step size was 5 dB for the first four reversals and then decreased to 2 dB for eight reversals. The last eight reversals were averaged to produce a final threshold for each run.

Subjects had approximately 1 h of training before data collection began to ensure they understood the task. These training tasks involved listening to tones in quiet, tracking masker thresholds for a fixed-level tone using on- and off-frequency maskers, and tracking signal threshold with fixed level on- or off-frequency maskers, all of which are standard tasks in the current study. Learning was evaluated by comparing thresholds in these conditions. Thresholds were considered similar if they were within 3 dB of each other, and typically each task was completed 3–5 times. If the thresholds in the practice session varied by more than 3 dB consistently, the subject would either receive more training on that specific task until the thresholds were within 3 dB of each other, or they were asked to come back for another session which included training. Most subjects had consistent thresholds after the initial practice session. After training, each session of data collection was 1–1.5 h to prevent attentional fatigue. Each condition was tested at least twice per session, and thresholds are an average of the last two thresholds recorded for that condition. These final thresholds served as the data reported in the current study. Runs with a standard deviation (SD) greater than 5 dB were discarded from the overall averages and the condition was repeated thereafter. Data from each subject were collected for a minimum of one session for each psychoacoustic task, and additional sessions were conducted if large variability or learning effects occurred. The order of presentation of conditions was interleaved across subjects. 

All statistical and post hoc analyses of gain reduction were performed using IBM SPSS 28 statistical software. Before conducting any statistical tests, all datasets were checked for normality using the Shapiro–Wilk test of normal distribution and corresponding normal Q-Q plots. With this test, any subset of data tested for this assumption with a p-value equal to or greater than 0.05 would meet the criterion to assume normal distribution. Aside from data from two subjects (S10 and S16), whose off-frequency masked threshold shifts were considered statistical outliers and therefore excluded from the analysis and overall averages, data from all other subjects met the assumption of normality (N = 17). A statistical outlier in this case was defined as any value that lies outside 1.5 times the interquartile range (i.e., above or below the 75th and 25th percentiles, respectively). All statistical tests in the current study met the assumption of normality.

### Results

#### Threshold Shifts with Elicitor Duration

**Fig. 2:**
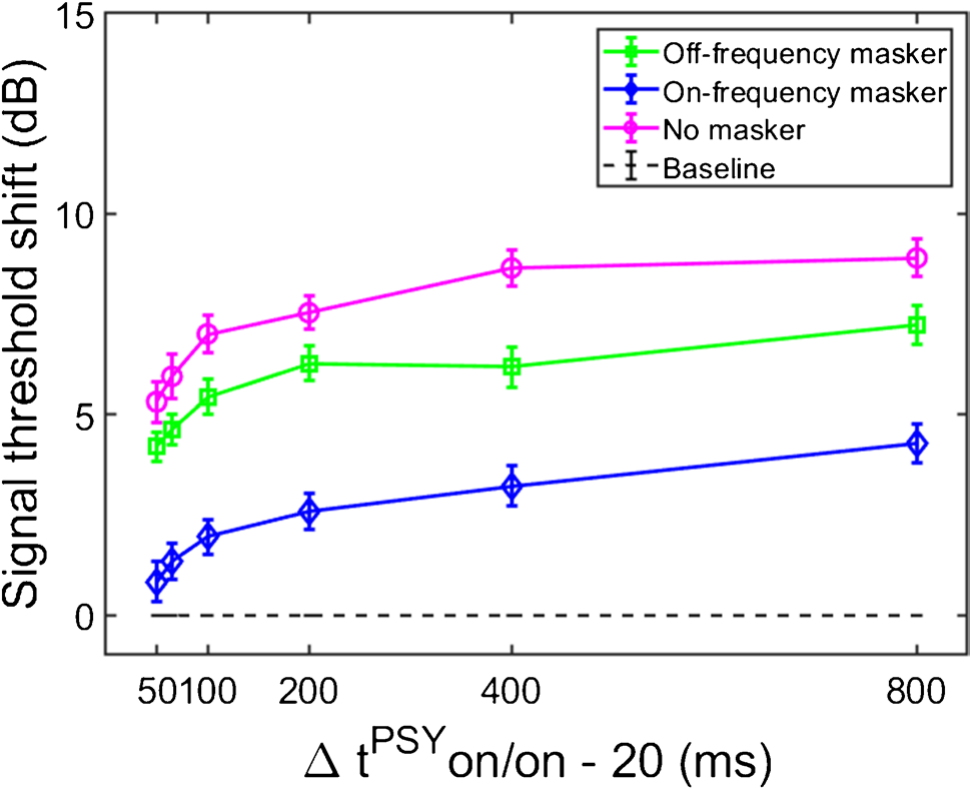
Average signal threshold shifts as a function of elicitor duration at 2 kHz (N = 17). Symbols represent listening condition, while the horizontal dashed line represents the reference condition with no elicitor. The baseline for the masker present conditions is signal threshold with a fixed level masker that shifted the signal by 5 dB, whereas quiet threshold of the signal served as the baseline for the no-masker condition. Vertical error bars indicate standard error of the mean (SEM)

The average threshold shifts (N = 17), as a function of elicitor duration, for the masker present and masker absent conditions are shown in Fig. 2. The dashed horizontal line at zero represents the baseline condition: signal threshold with a fixed masker for the masker present conditions, and quiet threshold of the signal for the no-masker condition, respectively. This arrangement allows us to compare the shifts in threshold by the elicitor across all conditions within the same figure. These thresholds were highly consistent across trials for all subjects, and were always within 2–3 dB of each other in the overall average. Additionally, the signal thresholds for the fixed on- and off-frequency masked conditions (no elicitor) were within 3 dB of each other for each subject, thereby confirming equivalent masking of the signal. These thresholds, as well as quiet thresholds, and masker thresholds for the signal at 5 dB SL, are reported for each subject in Supplementary File 2; Supplementary Table 1. Supplementary Table 1 also provides the total gain at the signal frequency for each subject by calculating the difference between the off-frequency and on-frequency masker threshold for the signal at 5 dB SL.

The overall qualitative pattern of the group data indicates that threshold shifts with an off-frequency masker (square symbol) are larger than threshold shifts with an on-frequency masker (diamond symbol). This pattern is consistent with gain reduction rather than excitatory masking. Similar to the large threshold shifts in the off-frequency masked condition, the growth function of the masker absent condition was also larger than the on-frequency masked condition. Both of these observations in the average data were tested statistically and are reported in the current section.

A two-way 3 × 6 repeated measures ANOVA was conducted to determine if the mean signal threshold shifts (dependent variable) significantly varied with the independent variables: masker type (off-frequency, on-frequency, and no masker) and elicitor duration (50, 65, 100, 200, 400, and 800 ms), including potential interaction effects between independent variables. The results are summarized here. The analysis indicated that there was a significant main effect of masker type (F(2,32) = 56.269, p < 0.001, η^2^ = 0.779). Bonferroni corrections revealed statistically significant differences in threshold shifts between the off-frequency masked condition and the on-frequency masked conditions (p < 0.001), with an average difference of 3.29 dB (SD = 0.30 dB) between the two conditions across elicitor durations. This result is consistent with gain reduction, and is not consistent with excitatory masking [34, 51]. A significant difference was also observed between the masker absent and on-frequency masked conditions, with an average difference of 4.86 dB (SD = 0.36 dB) between the conditions across elicitor durations (p < 0.001). A significant difference was also observed between the off-frequency and masker absent conditions, by an average of 1.58 dB (SD = 0.49) between the conditions across elicitor durations (p = 0.019). While this difference is relatively small, this finding differs from a recent study where the average difference between the off-frequency and masker absent gain reduction growth functions with elicitor duration, measured at 4 kHz, was non-significant [34]. However, in that study, the off-frequency masked threshold shifts were non-significantly larger than the masker absent condition (by an average of 2.12 dB across listening conditions). It is unclear why the off-frequency and masker absent conditions differed in threshold shifts between the studies, but there were differences in signal frequency used (2 kHz in the current study, and 4 kHz in the previous study), and the total sample size (19 total in the current study, and 9 total in the previous study), and individual differences in the subject responses across the two studies.

Next, a significant main effect of elicitor duration was found (F(2.823,45.168) = 8.865, *p* < 0.001, η^2^ = 0.786), indicating that gain reduction magnitude estimates generally increase with elicitor duration. For both the off-frequency and masker absent conditions, the average threshold shifts increase by about 1 dB or less with doubling of the elicitor duration. It’s important to note that the degrees of freedom in the F-values for elicitor duration are not integers. This is because sphericity could not be assumed for these data. The more conservative Greenhouse–Geisser critical F-value was reported instead, which helps correct for violation of sphericity. No interaction was found between the masker type and elicitor duration (F(10,160) = 1.425, p = 0.174, η^2^ = 0.082). Signal threshold shifts in the on-frequency condition followed the same pattern with elicitor duration as in the off-frequency and no masker conditions, but with smaller shifts in signal threshold. While it is expected that the on-frequency masker and signal are affected approximately equally by the elicitor (i.e., since the reduction of gain affects them equally at characteristic frequency), it is possible that the level of the signal is reduced below audibility with increased elicitor duration (see [34]).

Taken altogether, elicitor-induced threshold shifts were significantly larger for both the off-frequency and no-masker conditions compared to the on-frequency masked conditions. Therefore, the off-frequency and no-masker conditions are interpreted as gain reduction effects as has been done in prior studies [34, 51, 54].

#### Time Constants of Gain Reduction

The next step was to estimate time constants from the gain reduction data in the prior section. While all three conditions reflect gain reduction, many of the data points in the on-frequency masked condition are not significantly different from the baseline condition. The elicitor produced significantly larger effects for both the off-frequency masked and masker absent conditions (Fig. 2), therefore these two conditions were used to estimate the time constants. An inverse exponential function was fit to individual and group data from the previous section, and an overall time constant (τ) and a corresponding variance accounted for (R^2^) value were estimated. The formula for this function is, Y(t) = Y_max_(1 – e^−t/τ^) which estimates the time at which approximately 63% of the maximal effect of the growth function is achieved. In this formula, Y represents the event or response of interest, t represents elapsed time, and τ represents the time constant. Time constants estimated with this function have previously been used in human psychoacoustic and physiological measures of temporal overshoot [37], an effect that has been suggested to be related to gain reduction possibly via the MOCR. We have also used this function to fit psychoacoustic forward masking gain reduction with elicitor duration [34]. With respect to our psychoacoustic experiments, Y is the signal threshold shift when an elicitor is added (i.e., gain reduction). As explained in the psychoacoustic Stimuli section, both the onset delay of the MOCR (20 ms) and the delay between the offset of the elicitor and the onset of the signal (20 ms) were accounted for in the time constant estimations (Δt^PSY^ on/on - 20 ms). Therefore, the equation (Δt^PSY^ on/on - 20 ms) represents t in the time constant formula above, and equates to the elicitor duration for the psychoacoustic experiment.

**Fig. 3:**
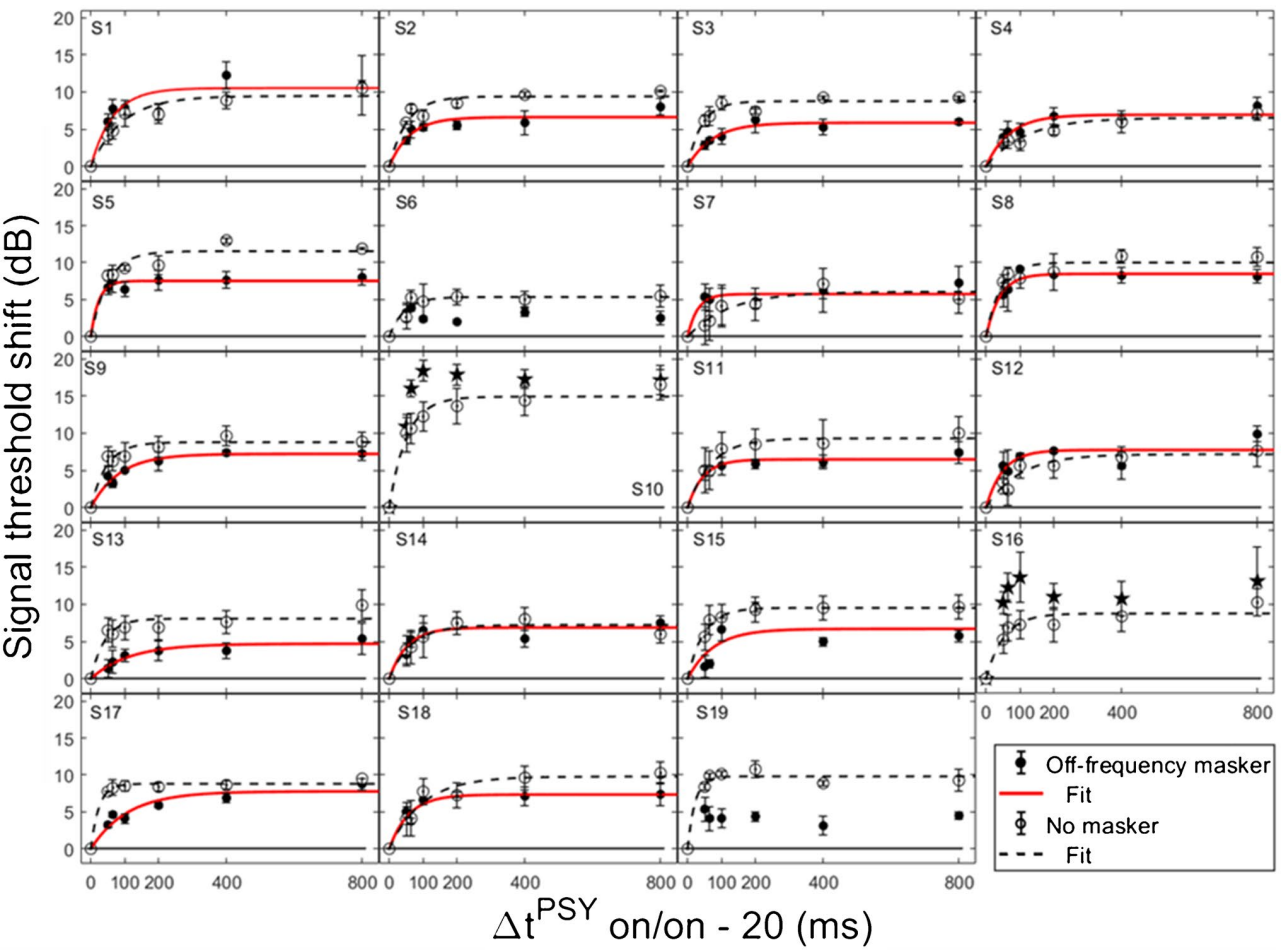
Individual subject signal threshold shifts as a function of elicitor duration. Filled symbols represent the off-frequency masked data, and open symbols represent the no masker data. On-frequency data are excluded because the off-frequency and no masker conditions provide the largest threshold shifts consistent with gain reduction (see Fig. 1), as well as for visual clarity. The solid and dashed curves are the exponential curve fits for the off-frequency and no masker conditions, respectively. SDs of signal thresholds are indicated by the error bars

**Fig. 4:**
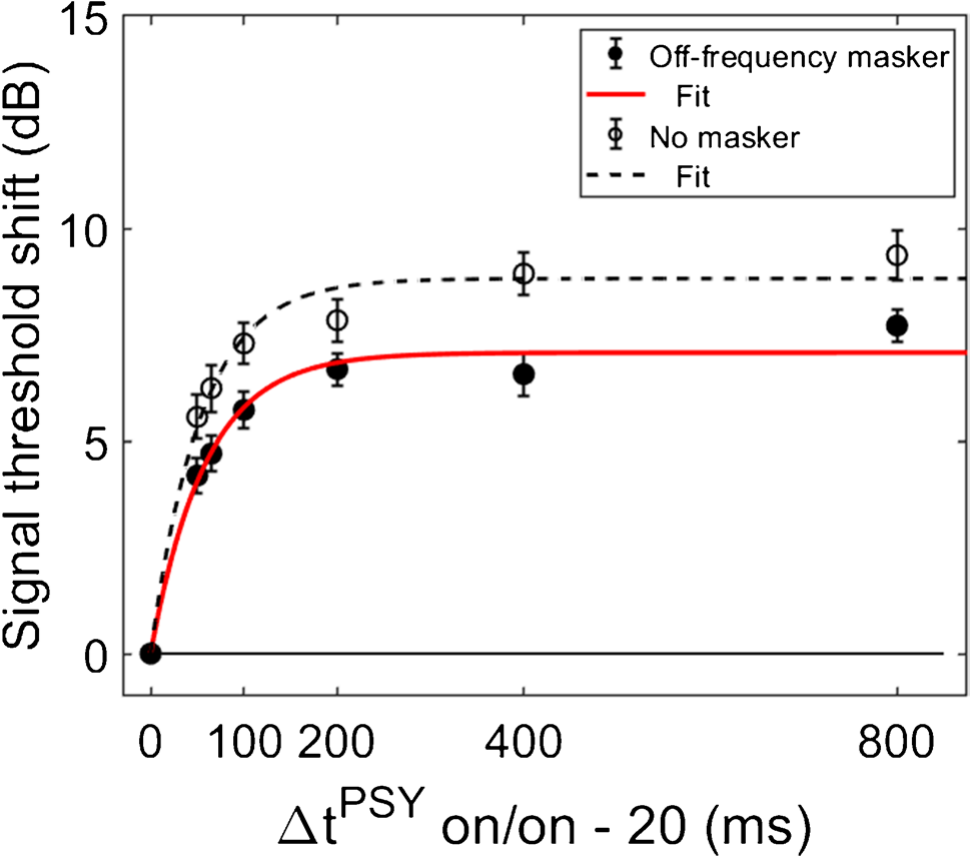
Average fitted signal threshold shifts, estimated from the data in Fig. 3 (also identically labeled), are shown as a function of elicitor duration. All individual data met the criteria for inclusion in the no masker average (N = 19). However, statistical outliers (S10 and S16) and data from subjects with no individual fits (S6 and S19) were excluded from the average off-frequency (N = 15) condition. SEM of signal thresholds is indicated by the error bars

In order for individual time constants to be included in the group averaged time constant, at least 60% of the variance in the data needed to be accounted for in the individual fit. Figures 3 and 4 contain individual and averaged data with curve fits, respectively. In Fig. 3, cells are labeled by subject number. The dashed horizontal line at zero in each cell represents the baseline condition: the signal threshold with a fixed masker for the off-frequency masked condition, and the quiet threshold for the signal for the no-masker condition, respectively. In addition to the data points in the time constant estimation, a point was added at zero on the abscissa and ordinate to represent the baseline condition as well as to ground the model. Closed circles denote off-frequency masked conditions, while open circles represent masker absent conditions. The fitted curve is solid for the off-frequency masked conditions and dashed for masker absent conditions. Off-frequency masked data for two subjects (S10 and S16) were considered outliers and were excluded from the statistical analysis, and are denoted by filled star symbols instead of filled circles, respectively. The individual time constants range from approximately 21 to 122 ms, and are shown in Table 1. Despite the range of time constant values for the individual data, all of the time constants were fairly short. For many of these individual functions, the maximal (Y_max_) or near maximal threshold shifts occurred for the 200-ms elicitor duration. The average time constant for the off-frequency masked conditions was 62.78 ± 6.62 ms (R^2^ = 0.92), and the average time constant for the no masker conditions was 61.72 ± 6.79 ms (R^2^ = 0.95), essentially equivalent results between conditions (Fig. 4). The buildup effect in the average data is near-maximal within approximately 200 ms of elicitor activation. Lastly, the exponential function fit the individual and the average behavioral data quite well, with over 90% of the variance accounted for the average fits and for the majority of the individual fits.

**Table 1:** Individual subject and averaged time constants from the psychoacoustic gain reduction experiments with corresponding R^2^ values for each condition. Time constants (***τ****)* units are expressed in milliseconds, and the ± error in the average is the SEM of the individual subject time constants. Asterisks (*) indicate that the data were not included due to being statistical outliers, identified based on the assumption of normal distribution before statistical testing. The ‘- ‘ symbol indicates that a time constant could not be estimated from the data. ‘N’ indicates the total number of subjects used in calculating the average time constant

Subjects	Off-frequency (Avg: N = 15)		No-masker (Avg: N = 19)	
	τ	R^2^	τ	R^2^
S1	64.16	0.89	92.60	0.95
S2	60.17	0.91	52.36	0.95
S3	73.13	0.97	41.51	0.97
S4	68.41	0.92	115.70	0.93
S5	23.14	0.97	49.68	0.95
S6	-	-	44.01	0.92
S7	29.04	0.84	122.40	0.91
S8	40.24	0.97	41.34	0.95
S9	82.75	0.97	46.09	0.96
S10	38.00*	0.96*	51.29	0.98
S11	44.61	0.97	68.56	0.97
S12	49.11	0.85	98.78	0.94
S13	113.60	0.94	40.74	0.91
S14	52.17	0.91	65.63	0.95
S15	76.21	0.69	47.18	0.99
S16	21.28*	0.95*	52.13	0.94
S17	107.60	0.93	23.75	0.99
S18	57.31	0.95	97.19	0.95
S19	-	-	21.69	0.97
Average	62.78 ± 6.62	0.92	61.72 ± 6.79	0.95

Not all threshold shift growth functions increase monotonically. In some individual cases, an oscillating effect was observed where the functions slightly increased and decreased with elicitor duration. These cases include: the off-frequency condition (S1, S3, S6-8, S10, S12, S14-16, S19), and the no masker condition (S2, S3, S8, S19). This oscillating effect has been documented previously with on-frequency tonal elicitors [64] and broadband noise elicitors [34, 58]. This phenomenon has been modeled as the elicitor turning down the gain at the signal frequency place and decreasing its own effectiveness as the elicitor duration increases [64]. An elicitor with reduced effectiveness can result in an improvement in signal threshold. Consistent with this interpretation, this oscillation is also evident with elicitor duration in some OAE data [23].

## Experiment 2: OAE MOCR Effects as a Function of Broadband Elicitor Duration

The second experiment measured the effect of ipsilateral broadband elicitor duration on transient-evoked OAEs (TEOAEs). Changes in the TEOAE magnitude as a function of elicitor duration were used to estimate time constants of MOCR gain reduction. All subjects that participated in experiment 1 also participated in experiment 2.

### Methods

#### TEOAE Measurements and Stimuli

TEOAE measurements were made with an ER-10X Extended-Bandwidth Acoustic Probe System (Etymotic Research), utilizing integrated forward pressure level (FPL) systems. All stimuli were generated with two TDT (Tucker-Davis Technologies) RZ6 programmable DSP processors, amplified by two separate TDT HB7 headphone drivers, and fed to the ER-10X probe. The ER-10X probe was calibrated by determining the Thévenin-equivalent characteristics of the sound sources [65], necessary to determine the acoustic impedance at a location in the ear canal. Thévenin-equivalent based FPL stimulus calibrations greatly correct for the effects of standings waves in the ear canal [66]. Thévenin-equivalent characteristics for the probe were estimated by measuring the acoustic response at the ER-10X microphone when the eartip was coupled to loads whose acoustic impedance values can be approximated using theoretical calculations. With the probe calibrated, the immittance properties of each subject’s ear canal (same ear tested from experiment 1) were checked for potential air-leaks or poor fitting of the probe. Air-leaks can cause changes of absorbance increasingly with the degree of the leak, especially at low-frequencies where air leaks reliably cause an increase of absorbance [67]. A criteria of low-frequency (0.2 kHz) absorbance was used to detect air leaks (less than 29%), or admittance phase greater than 44° [67]. Subjects chose a silent subtitled movie of their choice and were instructed to relax, stay awake, and to remain as still as possible during data collection.

**Fig. 5:**
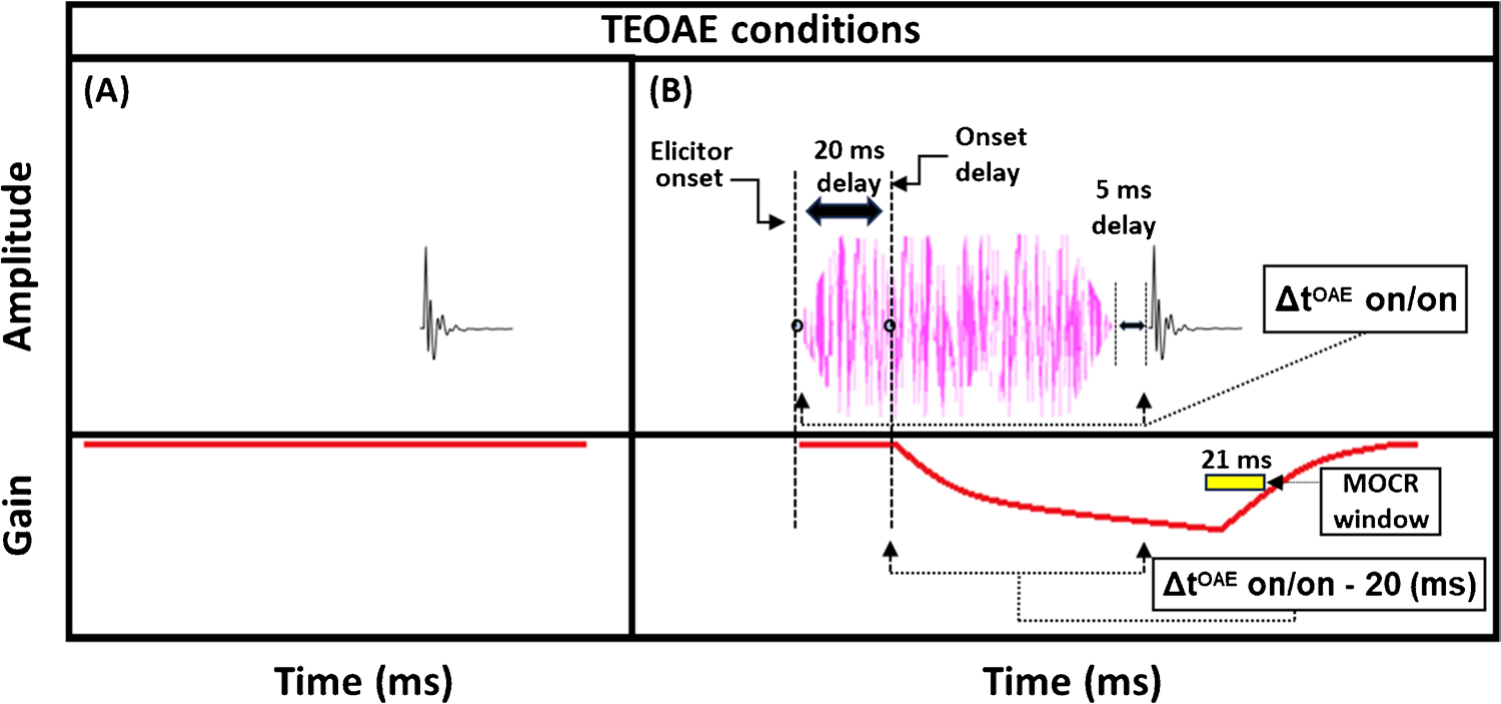
Schematics of the stimuli used in the TEOAE experiment. Accompanying the stimuli are estimations of the time course of gain reduction (bottom portion in each panel). **A** Baseline TEOAEs were measured with the click presentation alone, and always presented with the same temporal portion within the buffer window. **B** Elicitor durations included 50, 100, 200, and 400 ms. To account for the onset delay (bottom), the total time of elicitation starts 20 ms after elicitor onset and ends at the onset of the click (Δt^OAE^ on/on - 20 (ms)). Because the delay from the elicitor to the click (5 ms) is shorter than the onset delay (20 ms), ‘t’ is equal to the elicitor duration minus 15 ms for each data point. The yellow box in the bottom of panel **B** denotes a temporal region post-click at which the MOCR effects (ΔTEOAE) are measured from (~ 4 to 25 ms window)

TEOAEs were measured with 54 dB peak-equivalent FPL (peFPL) click probes with a flat incident power spectrum spanning approximately 0—10 kHz. This click level reliably produced emissions well above the noise floor and was likely too low to elicit the MOCR or MEMR, as discussed below. Previous studies examining MOCR effects on the TEOAE IO function have reported elicitor induced changes in emission magnitude at similar click levels (54 dB peak-equivalent SPL, [46]; 55 dB peak-to-peak FPL, [68]). These moderate click levels lie on the lower, more linear portion of the cochlear IO function, where MOCR effects tend to be larger compared to those observed at higher click levels (see Supplementary File 1 – Supplementary Fig. 1D; [46, 68]. The elicitor was a 50-dB FPL pink broadband noise (0.25–8 kHz), including 5-ms cos^2^ onset/offset ramps. Elicitor durations were 50, 100, 200, and 400-ms, and the elicitor always preceded the probe click by 5 ms (see Fig. 5). Contrasting from the psychoacoustic experiment, which used a 20 ms delay between elicitor offset and signal onset, the TEOAE experiment employed a shorter delay to significantly reduce total data collection time (saving ~ 15 min of total acquisition time). For the same reason, the 800 ms elicitor used in the psychoacoustic experiment was not included in the TEOAE measurements. The shorter delay between the elicitor and the click in the TEOAE measures also made it more likely that gain reduction had not yet started to decay.

Both the click and the elicitors were generated with a sampling rate of 48,828.125 Hz. Five different stimulus configurations [click alone (baseline) or click + elicitor] were presented in a sequential block format, equating to a single “set”. Each listening condition presentation within a set was followed by 2 s of silence to allow any potential efferent effects to decay back to baseline. Sixteen total sets were collected and stored as a single “array” of data. Eight total arrays of data were collected from each subject. After the discarding function in the program removed microphone or amplifier artifacts from each individual block for a given condition (the first 8 out of 40 responses), a total of 512 responses for each listening condition remained in an array. Thus, completing the experiment equated to an overall estimate from 4096 responses in the average for each listening configuration. Total data collection time equated to ~ 4–4.5 h, collected over 2–3 lab visits. In-ear probe calibrations were performed after every two arrays of data were collected to ensure that the probe had remained fitted during the testing session. The total duration of a single stimulus presentation (i.e., a single buffer) was always fixed at ~ 447 ms, including: 405 ms from the onset of the stimulus window to the onset of the click, and ~ 42 ms post-click. The time-window for measuring MOCR effects were recorded in a ~ 21 ms window, which started at time 3.93 ms post-click and ended 24.90 ms post-click (to remove the click acoustic waveforms and interfering high-frequency energy evoked from the click). Therefore, the only adjustment between stimulus conditions was the onset of the elicitor relative to the onset of the stimulus presentation window, which was dictated by the elicitor duration. A schematic of the click + elicitor condition is shown in Fig. 5B.

MOCR effects on TEOAEs produced by an elicitor can be analyzed by time-windowing the response following the presentation of the click probe (e.g., [26, 69]). These effects were quantified in two ways: 1) the change in the TEOAE magnitude alone (ΔTEOAE_m_), and 2) the change in the TEOAE that accounts for both magnitude and phase (ΔTEOAE_m+p_). For reference, in prior studies, ΔTEOAE_m_ has been expressed as Δ|TEOAE|, while ΔTEOAE_m+p_ has been expressed as |ΔTEOAE| [25, 70], and other studies have used similar metrics for the same quantities [56, 68]. Our data was collected in the time domain using a median average of the TEOAE waveforms, which preserves the temporal envelope of the response while reducing sensitivity to artifact-related outliers. A single Fourier transform was calculated from the averaged waveform. Then, the power spectral density for each listening condition was estimated using the multitaper method in MATLAB (MathWorks, Inc., Natick, MA). The difference between the click alone and the click + elicitor conditions is used to calculate the ΔTEOAE_m_ values. To account for both magnitude and phase in the TEOAEs, the vector difference of the ear canal sound pressure to the click alone condition (P_baseline_) and for the click + elicitor (P_elicitor_) conditions was calculated. The power spectral density of this value is the ΔTEOAE_m+p_ for a given listening condition. These metrics have been used in prior studies measuring elicitor induced MOCR effects on SFOAEs [25], and TEOAEs [26], both of which share a common reflection generation mechanism [71]. The ΔTEOAE was calculated for a 1/3rd-octave band centered at 2 kHz, a frequency range that was chosen to match the signal frequency in the psychoacoustic experiment. This analysis bandwidth has been used in previous TEOAE-based MOCR experiments [55]. Next, TEOAE-based MOCR time constants were estimated by fitting the data using the same function used in the psychoacoustic experiment. The parameters in this experiment have previously been described in further detail (see [72]), and were used in our recent work [48].

The resulting TEOAE_m_ data in this frequency range were typically 20–30 dB above the noise floor, noise floors ranged from approximately −30 to −10 dB SPL over the same frequency range. Therefore, all subjects in the current study had reasonable responses and their data are reported here. Changes in the TEOAE phase by the elicitor without accounting for magnitude changes (ΔTEOAE_p_) were also conducted in the current study. ΔTEOAE_p_ was quantified by calculating the average phase shift in the TEOAE spectrum between elicitor-present and baseline conditions. This was done within a 1/3-octave frequency band centered at 2 kHz, where the mean phase difference (in degrees) was computed across frequency bins. However, only small changes in phase angle occurred (~ 0.7*°* for the shortest duration elicitor and ~ 2.3° for the longest duration elicitor), with an overall non-significant positive phase shift (phase lead) with elicitor duration at the group level (Supplementary File 3; Supplementary Fig. 2 for details). However, at the individual subject level, some subjects had positive phase shifts while others had negative phase shifts with elicitor duration, and nearly all these shifts were within the margin of zero change in phase. TEOAE measures and data analysis, and hardware/software calibrations were made using a PC with custom MATLAB software. All measures described were recorded in a single-walled booth.

An additional analysis was used to determine if the MEMR was activated during the TEOAE experiments. Elicitor-induced absorbed-power changes were obtained concurrently in the TEOAE experiment by time-windowing the measurement to a few samples surrounding the input click. Significant changes to the input click level (> 0.1 dB) by the elicitor would indicate potential MEMR activation [26]. This was estimated by taking the difference in absorbed power of the click for the condition with the longest elicitor duration (400 ms) and the condition in which the click was presented alone. None of our subjects had absorbance values above 0.1 dB, further indicating that it was highly unlikely that the MEMR was active during the experiments (see [72]).

While we were able time window to the response to the acoustic click and to the MOCR window, we were unable to window the response to the latter portion of the buffer beyond these windows (for the current study, approximately ~ 26 to ~ 42 ms post-click) to analyze for the potential presence of synchronized spontaneous emissions (SSOAEs). SSOAEs are emissions that can be evoked by transient stimuli (i.e., clicks), and can become entrained to the evoking stimulus which contaminates the TEOAE amplitude and phase. SSOAEs can occur at specific frequencies, similarly to spontaneous otoacoustic emissions (SOAEs), and occur with longer delays than the TEOAE (typically ~ 20 ms post-click), which is why the presence of SSOAEs are typically analyzed by time windows after the MOCR window in TEOAE MOCR paradigms [26, 68]. We were unable to analyze SSOAEs because the TEOAE data were stored as an average response up to the end of MOCR window, and responses to time periods beyond this were not stored in the final data array. Similarly, we did not test our subjects for the presence of SOAEs. SOAEs are emissions that occur without sound stimulation at narrowband frequencies and can interfere and contaminate the TEOAE measurements. Analyses for both SSOAEs and SOAEs would be ideal in our future work, and is a potential limitation to the current study.

### Results

#### Magnitude and Time Constants of Gain Reduction

**Fig. 6:**
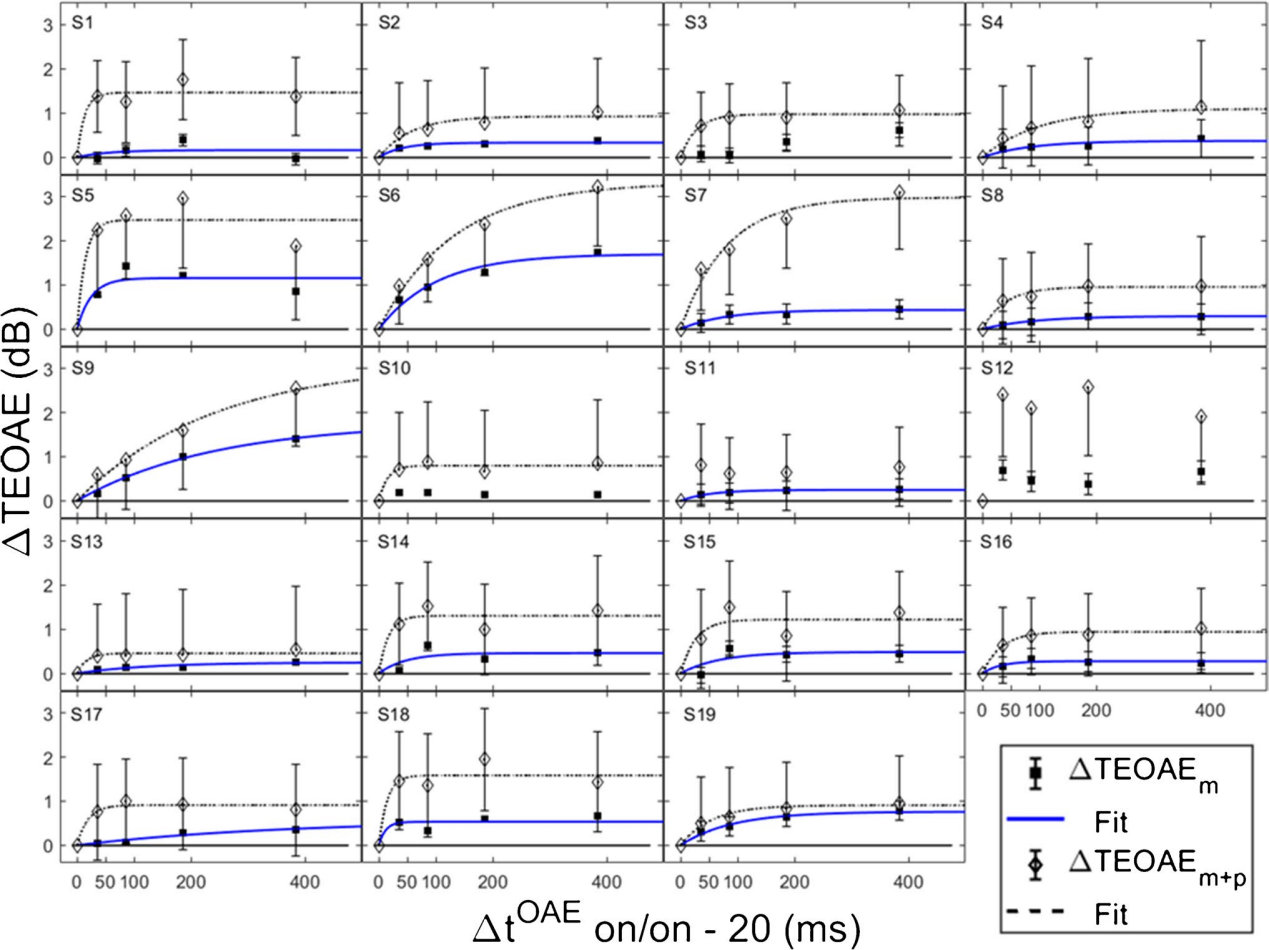
Individual subject fitted ΔTEOAE_m_ (filled squares) and ΔTEOAE_m+p_ (open diamonds) data as a function of elicitor duration. Each cell contains data from a single subject, along with exponential curve fits (solid = ΔTEOAE_m_; dotted = ΔTEOAE_m+p_). SD is indicated by the error bars, some of which are single-sided for visual clarity. Most individual fitting resulted in large R^2^ values (> 90%). However, some subjects’ data had horizontal and sometimes oscillating patterns, and thus no time constants were estimated for these subjects

**Fig. 7:**
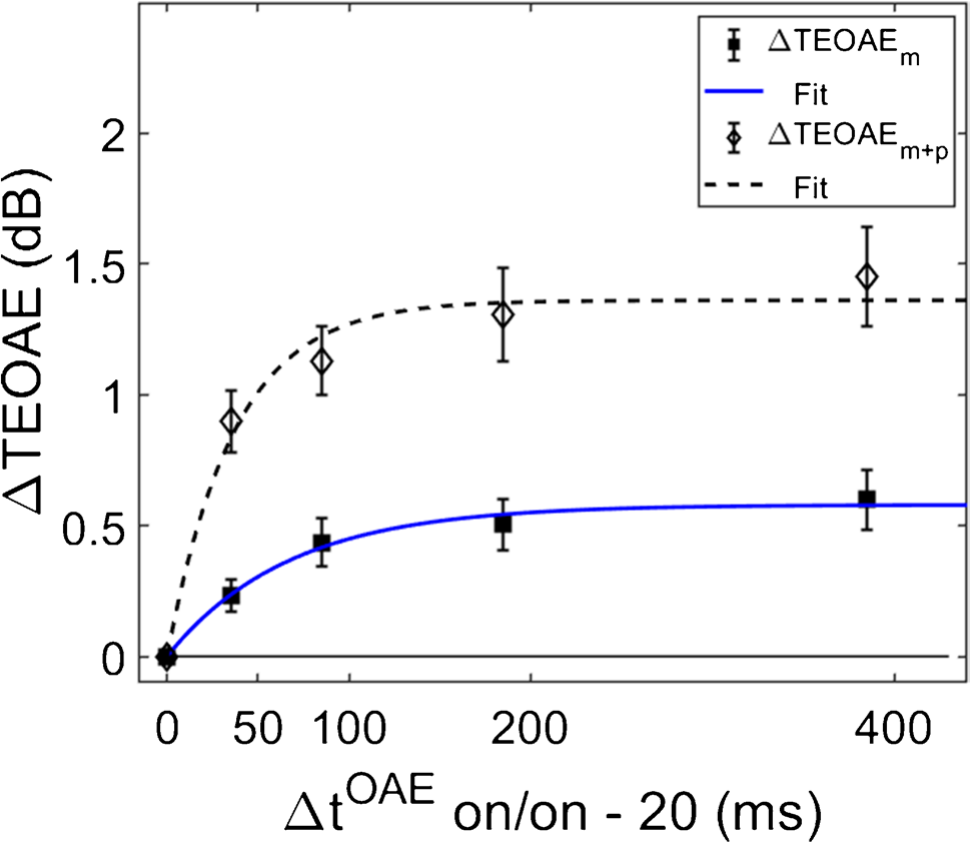
The average fitted ΔTEOAE_m_ (*N* = 15) and ΔTEOAE_m+p_ data with (*N* = 17), with average time constants of ~ 97 ms and ~ 53 ms respectively. SEM is indicated by the vertical error bars. The data were estimated from the data in Fig. 6, with identical labeling. The variance in both average datasets were well accounted for by the curve fitting (R^2^ values > 90%)

Both the magnitude and phase of the TEOAEs were affected by MOCR activation as a function of elicitor duration. Elicitor induced changes in the responses (ΔTEOAE) were quantified as ΔTEOAE_m_ and ΔTEOAE_m+p_. The fitted individual subject and average TEOAE data are shown in Fig. 6 and Fig. 7, respectively. Estimated time constants from these data are reported in Table 2. The time constant for the averaged ΔTEOAE_m_ data (N = 15) was 96.86 ± 23.24 ms (R^2^ = 0.91), with individual time constants ranging from ~ 12–367 ms. Data for four subjects (S1, S3, S10, S12) could not be fit due to horizontal (and sometimes oscillating) patterns, and thus their data were not included in the average data. Interestingly, some of the subjects with oscillating patterns in the TEOAE data as a function of elicitor duration also showed similar oscillating patterns in their psychoacoustic data (S1, S3, S10). The averaged ΔTEOAE_m_ effect size was within 1 dB across elicitor durations (Fig. 7). The time constant for the averaged ΔTEOAE_m+p_ data (N = 17, data for subjects 11 and 12 could not be fitted) was 52.80 ± 14.29 (R^2^ = 0.95), with individual time constants ranging from ~ 13–240 ms. The averaged ΔTEOAE_m+p_ effect size ranged from ~ 1–1.5 dB across elicitor durations (Fig. 7). Overall, the average elicitor induced shifts were larger for ΔTEOAE_m+p_ than for ΔTEOAE_m_ (Fig. 6 and Fig. 7). Small elicitor-induced MOCR changes on the TEOAE_m_ may be expected [26, 46, 68, 69], particularly with the level of the elicitor we used (50 dB FPL). Lastly, just as with the behavioral data, the exponential function fit the individual and the average TEOAE data quite well, with over 90% of the variance accounted for the average and most of the individual fits.

**Table 2:** Individual subject and averaged MOCR time constants from the TEOAE paradigm with corresponding R^2^ values for each condition. ***τ*** units are in milliseconds, and the ± error in the average is the SEM of the individual subject time constants. Asterisks (*) indicate that the individual data was not included in the averaged time constant due to a poor R^2^ (< 60%). The ‘- ‘ symbol indicates that a time constant could not be estimated from the data. ‘N’ is the subject total used in the average time constant.

Subjects	ΔTEOAE_m_ (Avg: N = 15)		ΔTEOAE_m+p_ (Avg: N = 17)	
	τ	R^2^	τ	R^2^
S1	54.96*	0.36*	13.05	0.94
S2	41.15	0.95	56.98	0.94
S3	-	-	27.87	0.99
S4	84.43	0.89	98.90	0.97
S5	24.82	0.87	14.29	0.91
S6	101.02	0.98	130.70	0.99
S7	77.87	0.97	80.58	0.98
S8	91.10	0.98	40.06	0.98
S9	240.08	1.00	239.90	0.99
S10	-	-	15.11	0.96
S11	51.62	0.97	-	-
S12	-	-	-	-
S13	124.60	0.93	16.82	0.95
S14	47.49	0.69	16.62	0.91
S15	68.19	0.73	26.26	0.84
S16	31.48	0.90	32.94	0.99
S17	366.90	0.96	18.98	0.97
S18	11.71	0.82	14.40	0.92
S19	90.50	0.98	54.28	0.99
Average	96.86 ± 23.24	0.91	52.80 ± 14.29	0.95

## Comparisons of Physiological and Psychoacoustic Measures of Gain Reduction as a Function of Elicitor Duration

Separate comparisons were conducted to test whether the responses in physiological measures of gain reduction (TEOAEs; experiment 2) could predict the responses in the psychoacoustic measures of gain reduction (experiment 1), as a function of elicitor duration. As stated in the Introduction, a correlation between these measures would be predicted due to a shared underlying mechanism (i.e., cochlear gain reduction via the MOCR). These comparisons were made using the two metrics we studied: the magnitude of gain reduction (ΔTEOAE and signal threshold shifts by the elicitor), and the time constants of gain reduction. Two combinations of comparisons were made: (1) ΔTEOAE_m_ and signal threshold shifts with the masker present (i.e., off-frequency masked), and (2) ΔTEOAE_m+p_ and signal threshold shifts with the masker absent.

The first combination, ΔTEOAE_m_ and threshold shifts with the masker present, are the most comparable metrics to those used in previous studies when comparing psychoacoustic and OAE measures of gain reduction. For example, the difference in masked thresholds from PTC’s with and without the presence of an elicitor have been compared to changes in OAE magnitude with an elicitor in previous studies [43–45]. As described in the Experiment 1 – Stimuli section, the masker present condition is consistent with gain reduction. The off-frequency masked condition in this study is the tail response of the PTC at the signal frequency place. The off-frequency masker is processed linearly at the signal frequency place and not affected by gain reduction by the elicitor. Thus, the difference in signal threshold between the off-frequency masked signal with a fixed level off–frequency masker with and without the elicitor provides an estimate of gain reduction at the signal frequency place [40, 42, 43]. The second combination, ΔTEOAE_m+p_ and threshold shifts with the masker absent condition, were compared due to their similarity in stimulus configuration, which include: an elicitor, followed by a short delay between the offset of the elicitor and the onset of the signal/probe, followed by the signal/probe (Fig. 5). Furthermore, the ΔTEOAE_m+p_ and the masker absent conditions produced the largest magnitude effects with elicitor duration (Fig. 4, Fig. 7). Because of the larger effects by the elicitors, we hypothesized that this comparison would reveal a strong predictive relationship between the two measures. Both combinations of comparisons were conducted using the ΔTEOAE data for elicitor durations of 50, 100, 200, and 400 ms.

Lastly, only a few studies have measured time-constants for both psychoacoustic and OAE measures of gain reduction in the same study [37, 48]. Those studies found similar time constants between their psychoacoustic and OAE measures at the group level. However, neither study made direct comparisons of data for individual subjects. Further, the study by Walsh et al. (2010) used a simultaneous-masking overshoot paradigm in both their behavioral and SFOAE experiments [37]. Gain reduction in the overshoot phenomena is likely mediated at least in part by the MOCR, but is also likely influenced by two-tone suppression [73]. Therefore, to our knowledge, the current study is the only work that estimates and directly compares the time-course of gain reduction psychoacoustically and physiologically in a forward-masking paradigm. Thus, the measures were hypothesized to reflect the same mechanisms while avoiding suppression.

### Results

#### Combination 1: Comparing Magnitude Effects

**Fig. 8:**
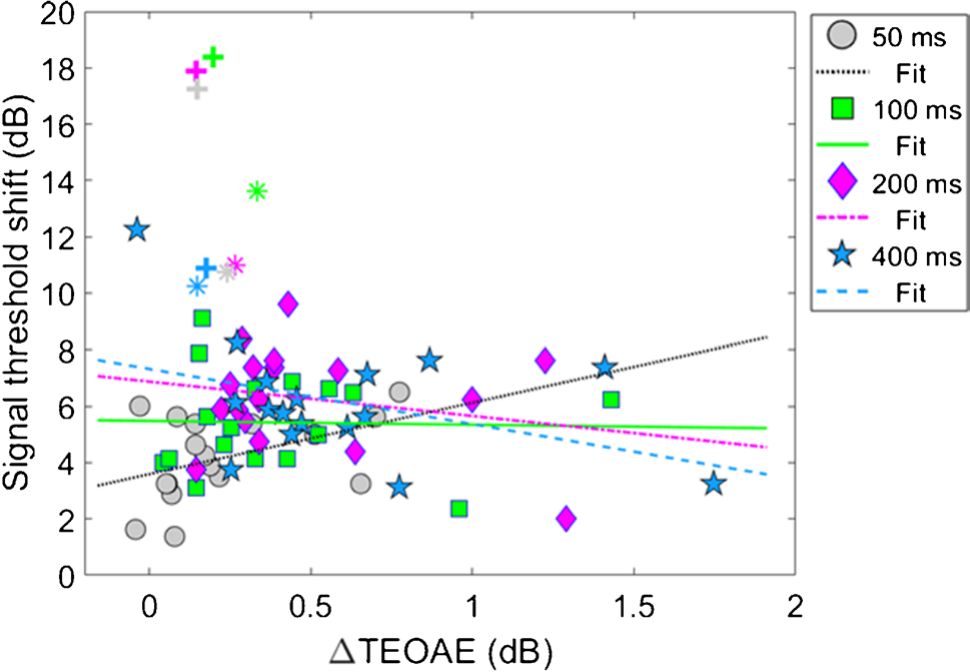
Group data on the relationship between ΔTEOAE_m_ and masker present conditions, as a function of elicitor duration (N = 17) (combination 1). Each coordinate represents changes in corresponding physiological and psychoacoustic responses for a single subject caused by the elicitor. Each cluster (coded by symbols and color) represents responses for a given elicitor duration, along with a fitted regression line (coded by line type and color), indicated in the legend. Two subjects’ data were considered outliers, shown by plus sign (S10) and asterisk (S16) symbols, respectively. While regression lines were fitted to each cluster (coded by line type and color), all corresponding R^2^ values were below 20%

Figure 8 shows the relationship between the ΔTEOAE_m_ and masker present conditions, as a function of elicitor duration (combination 1). Here, each coordinate represents the physiological (ΔTEOAE_m_) and psychoacoustic (off-frequency masked) response from a single subject. Coordinates are coded by symbols for each elicitor duration (50 ms = circles, 100 ms = squares, 200 ms = diamonds, 400 ms = stars). Thus, each subject will have four points in the figure. Linear regressions were fitted to each cluster of data for elicitor duration (50 ms = dotted, 100 ms = solid, 200 ms = dashed-dot, 400 ms = dashed). As noted earlier (see Experiment 1—procedure), data for two subjects (S10 and S16) were excluded from the analysis as these points were considered statistical outliers, and they are therefore reported here as plus sign (S10) and asterisk (S16) symbols, respectively. Therefore, for this comparison, each cluster and subsequent analysis represents data from a total of 17 subjects. Bonferroni corrections were applied to the alpha value in the correlational analysis to correct for multiple comparisons. The corresponding slopes (ΔdB/ΔdB), R^2^ values, and *p*-values for each regression line are reported here by elicitor duration: [50 ms] slope = 2.54, R^2^ = 0.19, and *p* = 0.077, [100 ms] slope = −0.14, R^2^ = < 0.001, and *p* = 0.92, [200 ms] slope = −1.22, R^2^ = 0.053, and *p* = 0.37, [400 ms] slope = −1.96, R^2^ = 0.16, *p* = 0.11. Overall, the linear fits were relatively flat (i.e., nearly zero slope) and were not strong predictors of the variance in the data (nonsignificant *p*-values and low R^2^ values). Therefore, no statistical comparisons were attempted across clusters using these regression statistics.

Instead, to account for individual subject variability in our data, a linear mixed-effects model (LMM) was used to determine the relationship between the ΔTEOAE_m_ (dB) and the threshold shifts in the masker present conditions (dB), as a function of elicitor duration. The dependent variable in this model was the signal threshold shift in the masker present data (i.e., gain reduction). Fixed effects in the model included ΔTEOAE_m_ (a continuous variable), and an interaction between ΔTEOAE_m_ and elicitor duration (a categorical variable: 4 levels; 50, 100, 200, 400 ms; ΔTEOAE_m_:Elicitor Duration). Individual subject responses were accounted for by making them a random effects variable in the model (1 | Subject). Random effects in an LMM are specified with the general structure (effect | grouping factor). The 1 in our expression denotes a random intercept, while the vertical bar (|) indicates the grouping factor across which the random effect varies. In our LMM, (1 | Subject) specifies that each subject has a different baseline level (random-intercept design), reflecting subject-specific starting points of gain reduction. This random-effects specification was applied across all other LMM analyses in our study. The resulting formula for this model is: Signal threshold shifts ~ ΔTEOAE_m_ + ΔTEOAE_m_:Elicitor Duration + (1 | Subject). Random slopes were not assigned to each subject response after initial testing of the model ruled out that assuming random slopes was inconsequential and would not be fitted in the maximal model (i.e., an overparameterization of random effects). A significant main effect would indicate that the overall change in the TEOAE_m_ predicted the overall shifts in the signal threshold by the elicitors. A significant interaction effect would indicate that the relationship between signal threshold shifts and ΔTEOAE_m_ significantly varied with elicitor duration (i.e., different slopes for each cluster).

All model coefficients were estimated using the restricted maximum likelihood procedure in the lme4 (version 1.4.1) library in *R* 3.6.3 [74] (R Core Team, 2020). Homoscedasticity and normality assumption tests were conducted on individual subject data, and both tests met the normality assumption based on their residual versus fitted plots. Statistical inferences about the fixed effects in the model were made with the F-approximation for the Scaled type-II Wald statistic [75]. Approximation with this statistic is a conservative approach in estimating false-alarm rates (type-I errors) compared to the Chi-squared approximation of the log-likelihood ratios, and has shown to be effective with smaller and complex datasets [76].

Model output and statistical values are reported in Table 3. Both fixed effects in the model, ΔTEOAE_m_ and the interaction of ΔTEOAE_m_ and elicitor duration, failed to meet statistical significance (p > 0.05). That is, the physiological changes (ΔTEOAE_m_) did not predict the psychoacoustic changes (signal threshold shifts) as a function of elicitor duration for this comparison. Furthermore, the non-significant interaction supports that the observed relationship between physiology and behavior did not change with elicitor duration. This result is apparent as the clustered groups are nearly completely overlapped (Fig. 8). One last finding is that the random effect variable to account for individual subject variance was lower in value than the residual error (unexplained variance) in the model, indicating that the outcome of the findings was not significantly influenced by the response variability across subjects.

**Table 3:** Summary of the linear mixed-effects model (LMM) detailing the relationship of ΔTEOAE_m_ and its interaction elicitor duration (independent variables) on signal threshold shifts (dependent variable). Type-II Wald approximations were used to generate F-statistics and p-values for the fixed effects. Neither of the fixed effect factors in the model were significant. Individual subject variability modeled as random effects were smaller than the residual error (uncontrolled variance), indicating subject variability played little-to-no role in the inferential statistics

	Model Output			Type-II Wald F-tests	
Fixed Effects	Estimate	Standard Error	t-value	F-statistic	p-value
Intercept	5.17	0.51	10.04	-	-
ΔTEOAE_m_	0.65	0.87	0.75	F(1,61.93) = 2.51	p = 0.12
ΔTEOAE_m_ : Elicitor Duration	0.98	0.90	1.09	F(3,50.98) = 0.61	p = 0.61
Random effects	Variance	Standard deviation			
By-subject intercepts	1.90	1.38	-	-	-
Residual	2.23	1.49	-	-	-

#### Combination 1: Comparing Time Constants

**Fig. 9:**
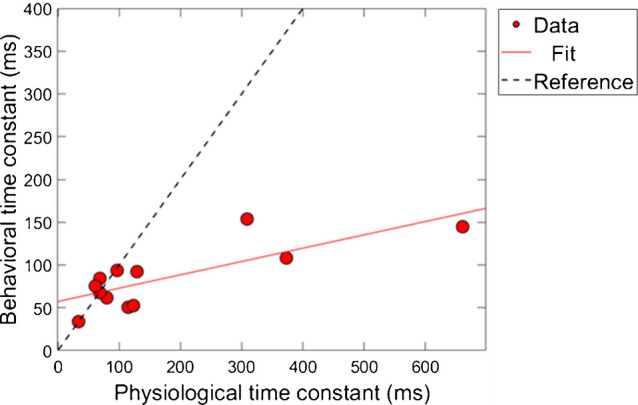
The relationship between individual subject physiological and psychoacoustic-derived time constants, for ΔTEOAE_m_ and masker present conditions, respectively (N = 12) (combination 1). The dashed line is a reference indicating a 1 ms/1 ms slope (i.e., a perfect positive correlation). There was a strong, positive correlation between the time constants (R^2^ = 61.3% and *p* < 0.05; α = 0.05)

Next, a correlational analysis was conducted on the time constants of gain reduction for the conditions of combination 1 (ΔTEOAE_m_ and masker present). For time constants to be included in this analysis, the corresponding R^2^ values associated with the fit needed to be at least 60% or higher, as was indicated in earlier sections. Therefore, for a subject’s data to be included into the analysis, both of their fitted psychoacoustic and physiological data needed to meet this criteria. Figure 9 shows the results of this comparison. The axes are the time constant values in milliseconds, and each data point corresponds to a single subject’s data. The solid line is a linear regression fitted to the data. The dashed line is a reference indicating a hypothetical 1:1 ratio of time constants. Linear regression fitting showed a significant positive relationship between the physiologically and behaviorally measured time constants, with a slope of ~ 0.16 ms/ms, R^2^ = 61.3% and *p*-value = 0.0026 (p < 0.05; α = 0.05).

A few observations are made about the data and analysis. First, we note that the majority of time-constants are located on the bottom left portion of the plot, indicating that most of the time-constants were fairly short overall for both measures of gain reduction. However, there are a few data points that lie further outside of this cluster (i.e., a subject with a ~ 660 ms TEOAE time constant). A “leave one-out” method was used to determine if these longer time-constants played a role in the significance on the correlation between the two measures. By removing any of the points individually from the dataset, including the longest time constants (~ 660 ms), the correlation was still relatively large (R^2^ = ~ 58%) and significant (p < 0.05; α = 0.05) for all iterations of this method. In other words, there were no influential points in the analysis. This finding is in contrast from the magnitude results where no significant relationship was found between the physiological and psychoacoustic measures of gain reduction as a function of elicitor duration (Fig. 8).

#### Combination 2: Comparing Magnitude Effects

**Fig. 10:**
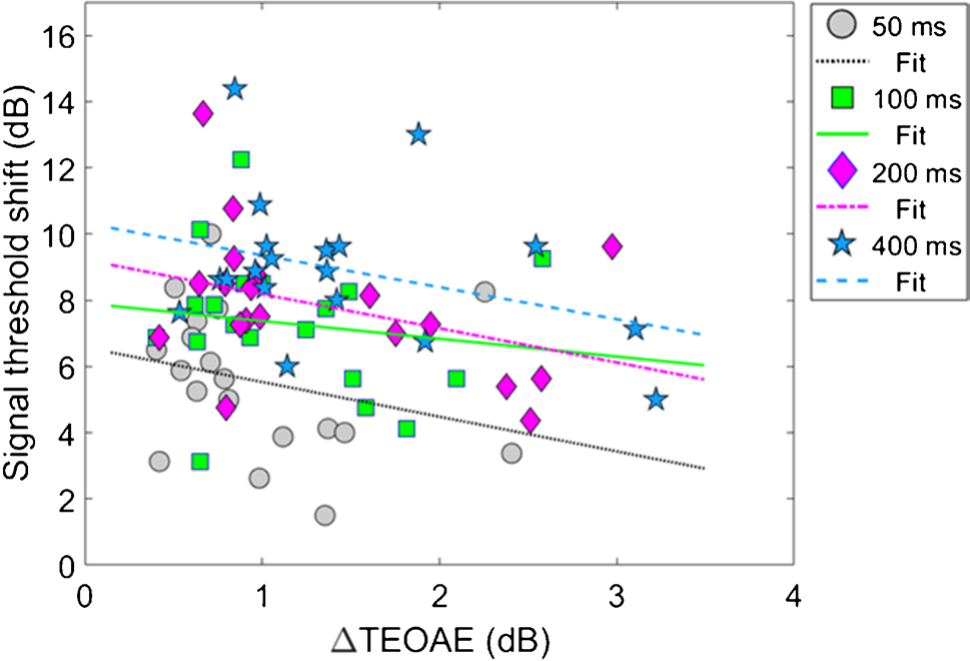
Group data on the relationship between ΔTEOAE_m+p_ and masker absent conditions (N = 19) (combination 2). This figure uses identical coding of symbols, line type, and color as in Fig. 8. Linear regression fitting of each cluster resulted in negative trends, all with R^2^ values below 20%. The clusters are stratified with increased elicitor duration

Next, comparisons were also made for the second combination of measures of gain reduction. Figure 10 shows the relationship between the ΔTEOAE_m+p_ and masker absent conditions, as a function of elicitor duration (combination 2). Figure 10 shares the same coding of symbols for the data and linear regression fitting as in Fig. 8. Each coordinate represents a single subject’s physiological (ΔTEOAE_m+p_) and psychoacoustic (no masker) response as a function of elicitor duration. For this comparison, each cluster and subsequent analysis represents data from a total of 19 subjects. Bonferroni corrections were applied to the alpha-value in the correlational analysis to correct for multiple comparisons. The corresponding slopes, R^2^, and corresponding *p*-values for each regression line are reported here by elicitor duration: [50 ms] slope = −1.05, R^2^ = 0.073, and *p* = 0.26, [100 ms] slope = −0.54, R^2^ = 0.021, and *p* = 0.55, [200] slope = −1.03, *R*^2^ = 0.14, and *p* = 0.12, [400 ms] slope = −0.97, R^2^ = 0.11, *p* = 0.16. All of the linear fits had similarly negative slopes, and were not strong predictors of the variance in the data (nonsignificant *p*-values and poor R^2^ values). Therefore, no statistical comparisons were attempted across clusters using these regression statistics.

A LMM was used to determine the relationship between the ΔTEOAE_m+p_ (dB) and the threshold shifts in the masker absent conditions (dB), as a function of elicitor duration. The dependent variable in this model was the signal threshold shift in the masker absent data. Fixed effects in the model included ΔTEOAE_m+p_ (a continuous variable), and an interaction between ΔTEOAE_m+p_ and elicitor duration (a categorical variable: 4 levels; 50, 100, 200, 400 ms; ΔTEOAE_m+p_:Elicitor Duration). Individual subject responses were accounted for by making them a random effects variable in the model (1 | Subject). The resulting formula for this model is: Signal threshold shifts ~ ΔTEOAE_m+p_ + ΔTEOAE_m+p_:Elicitor Duration + (1 | Subject). The random-intercept design was used while random slopes was not assumed, both for the same reasons as in the first comparison. Statistical analysis and inferences for the second combination were conducted identically to the first combination of data.

Model output and statistical values are reported in Table 4. Both fixed effects in the model, ΔTEOAE_m+p_ and the interaction of ΔTEOAE_m+p_ and elicitor duration, were highly statistically significant (p < 0.0001; α = 0.05). That is, the physiological changes (ΔTEOAE_m+p_) did relatively well at predicting the psychoacoustic changes (signal threshold shifts) as a function of elicitor duration for this comparison. Furthermore, the significant interaction supports that the relationship between the physiology and perception significantly differed with elicitor duration. This is apparent in Fig. 10 as the clustered groups were clearly stratified with elicitor duration, with the larger effects on the TEOAE and signal threshold shifts for longer duration elicitors. However, the observed relationship for this comparison was negatively correlated for all elicitor durations, which was not what we had originally hypothesized. One last finding is that the random effect variable to account for individual subject variance was significantly larger in value than the residual error in the model (by more than four times), indicating that the outcome of the findings was significantly influenced by the response variability across subjects. This indicates that including TEOAE phase in the responses greatly increased both the changes in the magnitude of the TEOAE by the elicitor, but also greatly increased the variability of the overall responses across subjects.

**Table 4:** Summary of the LMM detailing the relationship of ΔTEOAE_m+p_ and its interaction elicitor duration (independent variables) on signal threshold shifts (dependent variable) Type-II Wald approximations were used to generate a corresponding F-statistic and *p*-value for the fixed effects. Both fixed effects in the model were highly statistically significant. Individual subject variability was large compared to the residual error, indicating that subject variation in gain reduction played a significant role in the resulting inferential statistics. Significant effects are denoted with asterisks (*).

	Model Output			Type-II Wald F-tests	
Fixed Effects	Estimate	Standard Error	t-value	F-statistic	p-value
Intercept	8.09	0.62	13.02	-	-
ΔTEOAE_m+p_	−0.70	0.33	−2.12	F(1,64.16) = 19.69	p < 0.0001*
ΔTEOAE_m+p_: elicitor duration	1.94	0.19	10.43	F(3,54.73) = 37.90	p < 0.0001*
Random effects	Variance	Standard deviation			
By-subject intercepts	4.14*	2.04	**-**	**-**	**-**
Residual	0.80	0.89	**-**	**-**	**-**

#### Combination 2: Comparing Time Constants

**Fig. 11:**
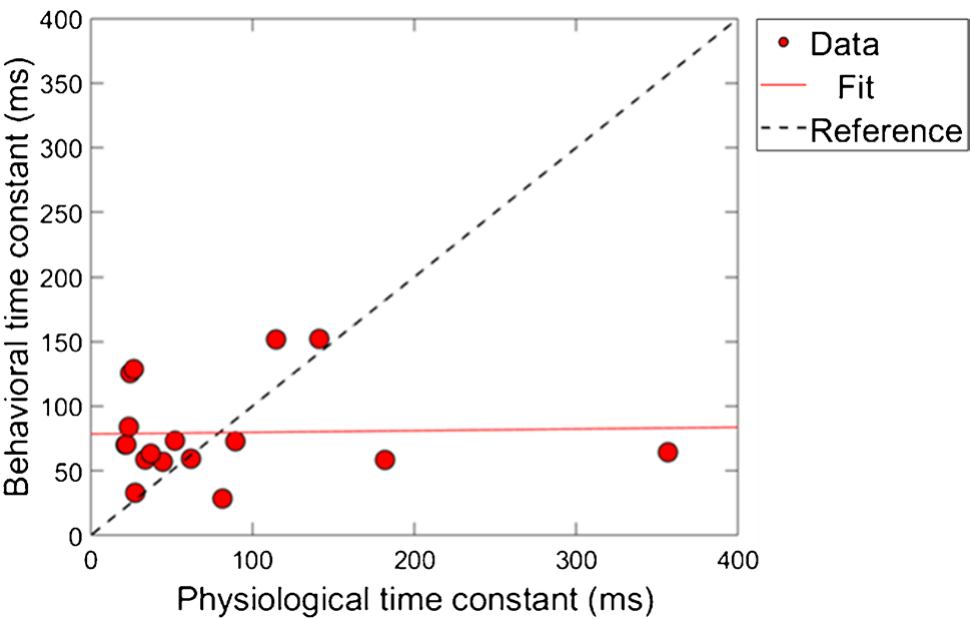
The relationship between individual subject physiologically and psychoacoustically derived time-constants, for ΔTEOAE_m+p_ and masker absent conditions, respectively (N = 17) (combination 2). The figure uses the same coding as Fig. 9. No correlation exists between the time constants (R^2^ < 1% and *p* > 0.05; α = 0.05)

Next, a correlational analysis was conducted on the physiological and psychoacoustic time constants of gain reduction for the conditions of combination 2 (ΔTEOAE_m+p_ and masker absent). The criteria for the data in this analysis was identical to combination 1. Figure 11 shows the results of this comparison, labeled identically to Fig. 9. Linear regression fitting showed a non-significant relationship between the physiologically and behaviorally measured time constants, with a nearly flat slope (0.013 ms/ms), R^2^ < 1% and *p*-value = 0.91 (p > 0.05; α = 0.05). Virtually no variation in the data can be explained by the linear fit. Just as in combination 1 (Fig. 9), the majority of time-constants are located on the bottom left portion of the plot, indicating that most of the time-constants were fairly short overall for both measures of gain reduction. We also conducted the “leave one-out” method to determine if any of the time-constants played a role in the relationship the two measures, and the no influential points were found in the analysis.

The two combinations of gain reduction measures resulted in complicated and contrasting findings. That is, for the comparisons made in combination 1, magnitude changes by the elicitor produced non-significant and nearly flat and sometimes negative regression fits (i.e., no correlation), while analyzing the time constants resulted in a significant and positive correlation. In contrast, for the comparisons made in combination 2, magnitude changes by the elicitor produced significant negative correlations, while analyzing the time constants of gain reduction resulted in no correlation.

## Discussion

This study investigated the effects of broadband elicitor duration on gain reduction using forward-masking psychoacoustic and TEOAE paradigms in adults with normal hearing. Direct comparisons were made between the measures of gain reduction, examining both their magnitude and time constants for individual subjects. We initially hypothesized that a positive correlation between physiological and psychoacoustic responses (i.e., magnitude effects), and between the physiological and psychoacoustic time constants of gain reduction would support the idea of a shared underlying mechanism—namely, gain reduction mediated by the MOCR. The methods of the current study were informed by previous psychoacoustic research employing tonal elicitors to characterize the temporal dynamics of gain reduction [41, 64]. These studies used on-frequency and off-frequency elicitors with durations ranging from 5 to 100 ms [41] or 10 to 150 ms [64], with a 4-kHz tonal signal. For most subjects, on-frequency elicitors produced maximal threshold shifts at durations of ~ 50 ms, beyond which the effects either plateaued or oscillated. In contrast, thresholds with off-frequency elicitors continued to increase with duration. Roverud and Strickland (2014) estimated time constants by applying a model that incorporated both a temporal integration window and a delay in the onset of gain reduction, yielding time constants ranging ~ 28 to 78 ms [64]. Given the maximal or near-maximal psychoacoustic gain reduction effect with ~ 50 ms on-frequency tonal elicitors, our previous studies adopted broadband elicitors of comparable duration. However, prior to the current study, the specific effects of broadband elicitor duration on psychoacoustic measures of gain reduction were largely underexplored. While this issue was introduced earlier, it is worth reiterating here: psychoacoustic studies have often employed broadband elicitors of longer durations (> 100 ms), or continuous elicitors, in an effort to maximize the MOCR response based on the time constants reported in OAE studies [23, 24]).

Psychoacoustic estimates of gain reduction ranged from ~ 4–7 dB for the masker present condition and ~ 5–9 dB for the masker absent condition, which increased with elicitor duration (Fig. 2). Physiologically, ΔTEOAE_m+p_ was larger than ΔTEOAE_m_ as a function of elicitor duration (Fig. 7), where ΔTEOAE_m+p_ showed changes of ~ 1–1.5 dB, while ΔTEOAE_m_ was typically less than 1 dB. The average psychoacoustic time constants were ~ 62–63 ± 7 ms (Fig. 4). Physiological time constants averaged ~ 53 ± 15 ms for the ΔTEOAE_m+p_ condition and ~ 97 ± 24 ms for the ΔTEOAE_m_ condition (Fig. 7). That is, when phase was accounted for in the TEOAEs, the physiological time constants closely matched the psychoacoustic time constants. However, when phase was omitted and only TEOAE magnitude changes were analyzed, the physiological time constants were nearly twice as long. Both psychoacoustic and physiological measures showed a buildup effect that increased with elicitor duration, reaching near-maximal levels within ~ 200 ms of elicitor onset. Exponential curve fitting effectively modeled both individual and group data, with most R^2^ values exceeding 90%.

While the psychoacoustic and physiological time constants were similarly short and consistent with cochlear gain reduction (possibly via the MOCR), our correlational analyses revealed that physiological responses were poor predictors of psychoacoustic responses across all elicitor durations. Most analyses showed non-significant, flat, or negatively trending relationships with elicitor duration when fitted by linear regression. This led to counterintuitive and mixed results when comparing elicitor-induced magnitude effects (ΔTEOAE and signal threshold shifts) and time constants of gain reduction. For instance, in terms of magnitude effects, ΔTEOAE_m_ did not significantly predict signal threshold shifts in the masker present condition, as a function of elicitor duration (comparison 1; Fig. 8). In contrast, ΔTEOAE_m+p_ significantly predicted signal threshold shifts in the masker absent condition, as a function of elicitor duration (comparison 2; Fig. 10). Interestingly, the relationships differed for the time constants of gain reduction: ΔTEOAE_m_ was significantly positively related predictive of signal threshold shifts in the masker present condition (comparison 1; Fig. 9), whereas ΔTEOAE_m+p_ showed a non-significant, flat relationship in the masker absent condition (comparison 2; Fig. 11). With multiple subjects not included in the correlational analysis due to poor fitting or considered as outliers, it unclear what this relationship would be with our original sample size (N = 19) or a larger subject pool as there is clear variation across subjects.

### Comparing the Time Course of Gain Reduction to Other Psychoacoustic and Physiological Measures

The psychoacoustic and physiological time constants of gain reduction found in the current study (< 100 ms) align with the fast time constants of MOCR buildup previously measured using OAEs in humans (60–100 ms) [23, 24, 29, 30]. Similarly short time constants, in the tens of milliseconds, have been observed in animal studies with electrical activation of the OCB, including at the level of the basilar membrane [32] and the auditory nerve [33].

The psychoacoustic magnitude and time course data in the current study closely resemble those in our previous work. Salloom et al. (2023) used the same psychoacoustic paradigm and elicitor characteristics but employed a 4 kHz signal frequency, compared to the 2 kHz signal frequency in the current study. The time constants were ~ 46 ms for the masker-present condition and ~ 78 ms for the masker-absent condition. In both cases, the buildup of threshold shifts was maximal or near-maximal within approximately 200 ms of elicitor activation [34].

One prior psychoacoustic study examined forward masking of a 6-kHz tonal signal using broadband noise maskers of varying durations (5, 10, 30, and 200 ms) [58]. One configuration included a 20 ms delay between masker offset and signal onset, identical to the no masker condition used in current study. Maskers were presented either 0 dB spectrum level (approximately 40 dB SPL) or 40 dB spectrum level (approximately 80 dB SPL). At 0 dB spectrum masker level, two of the four subjects exhibited maximal masking for the 30-ms masker, followed by reduced masking with the 200-ms masker-showing an oscillation pattern. The other two subjects showed no consistent change in masking effectiveness across masker durations (i.e., flat). At 40 dB spectrum masker level, two subjects exhibited increased masking with increasing masker duration, while the other two subjects showed plateauing responses for the 30 ms masker. Of the latter two subjects, one subject showed reduced masking at 200 ms, while the other showed a continued plateau. Taken together, these observed patterns—increased masking with masker duration, oscillatory masking effects, and early plateaus—are qualitatively consistent with trends seen in our psychoacoustic experiments. However, the masker durations tested in that study did not include intermediate durations between 30 and 200 ms, nor durations longer than 200 ms. As a result, it is difficult to determine whether the observed plateaus reflected the peak of the masking function or whether masking would have continued to change with longer masker durations.

The temporal properties of gain reduction observed in the current study align with findings from Walsh et al. (2010), who measured the “overshoot” effect using a psychoacoustic paradigm and a modified SFOAE paradigm (the nSFOAE) [37]. Overshoot refers to the phenomenon where a tonal signal presented at the onset of a masker is less detectable than when preceded by an additional sound, known as an elicitor [77]. In the Walsh et al. study, both paradigms used a 4-kHz tonal signal and a 400-ms broadband noise masker. The psychoacoustic measurements and SFOAE responses exhibited similar time constants of approximately 65 ms and 72 ms, respectively. Additionally, individual data revealed maximal or near-maximal buildup of the overshoot effect when the signal was delayed by ~ 100 ms from masker onset [37]. These temporal overshoot effects are similar to the time course of our psychoacoustic and TEOAE measurements, suggesting similar mechanisms (gain reduction possibly via MOCR). For clarification, in the Walsh et al. study, as the temporal gap between the elicitor and the probe signal increased, both the nSFOAE SNR and behavioral detection sensitivity improved, indicating that the MOCR was likely contributing to a greater reduction in cochlear sensitivity to the masker than to the signal. While overshoot is measured in simultaneous masking paradigms where MOCR activation may enhance detectability, the current study used forward masking, which involves an increase (worsening) in behavioral thresholds and a reduction in TEOAE amplitude.

Gain reduction effects with broadband elicitors often required longer durations (~ 150–200 ms) to reach maximal or near-maximal levels, with small but continued growth in most subjects (see Fig. 3). A previous study has shown a shorter time course for an on-frequency elicitor than for an off-frequency elicitor nearly an octave below the signal frequency [41]. Previous research has shown that broadband elicitors reduce cochlear gain [51] and broadens cochlear tuning [44]. As gain reduction broadens cochlear filters, the decrease in gain at the tip of the filter may be offset by the widening of the filter, making the effects of broadband elicitors similar to the off-frequency elicitor conditions used by Roverud and Strickland, 2010 [41]. The interplay between these factors likely explains the oscillation in signal threshold shifts and ΔTEOAE with elicitor duration observed in some subjects. These findings suggest that the time course of gain reduction depends on the specific characteristics of the elicitor used to evoke the effect including its frequency relative to the signal and its overall bandwidth [78]. The current study was limited to elicitor durations of 800 ms in the psychoacoustic experiment and 400 ms in the TEOAE experiment, and it is unclear if longer duration elicitors would have had any significant effect on the responses.

### Considerations for the Lack of Correlation Between Physiological and Psychoacoustic Gain Reduction

Consistent with the correlational results of the current study, previous research examining the relationship between psychoacoustic and otoacoustic measures of MOCR gain reduction has yielded mixed and often inconclusive results. Below, we describe studies that used metrics comparable to those in the current study, focusing on elicitor-induced magnitude changes on the response. We also discuss possible reasons for the inconsistent results across these studies and the current one. Studies comparing speech perception in noise tasks to changes in OAE levels are beyond the scope of this research and are not discussed here. Psychoacoustic tasks used in these comparisons are thought to reflect gain reduction or related mechanisms when an elicitor is present, including overshoot [37, 50], signal intensity discrimination [49], signal threshold in quiet [46], and PTCs [43, 44]. Most studies found no significant relationship between psychoacoustic and physiological measures [44–46, 50]. However, some reported significant positive relationships between the two measures [37, 43, 49].

Several factors may explain the mixed results regarding the relationship between psychoacoustic and physiological measures of gain reduction. One key issue is the considerable variability in methodologies and stimuli across studies. For example, some studies used simultaneous masking paradigms [37, 44, 50], which inherently involve two-tone suppression. This makes it difficult to disentangle the contributions of MOCR activation from those of two-tone suppression. Even when methodologies and stimuli were similar between studies, their overall results conflicted from one another (e.g., [37, 50]). Furthermore, the type of otoacoustic emission (OAE) paradigms also varied widely in these studies. Some studies measured changes in distortion-product OAEs (DPOAEs; [2f1-f2 amplitude]; [43, 44], while others focused on transient-evoked OAEs (TEOAEs; [45, 49]), stimulus-frequency OAEs (SFOAEs; [37, 50]), or a combination of TEOAEs and DPOAEs [46]. Importantly, DPOAEs do not share the same primary generation mechanism as TEOAEs and SFOAEs [71], raising questions about how these differences impact the results in those studies. Additionally, it is unclear how much of a factor OAE probe calibration methodology varied across these studies, as it can be a significant factor in the reliability of measurements [66, 67].

Most studies employed contralateral elicitors to activate the MOCR [43–46, 49], which avoids excitatory masking effects on the probe in the ipsilateral ear. In contrast, overshoot studies used ipsilateral stimuli [37, 50]. Ipsilateral elicitors produce significantly larger gain reduction effects psychoacoustically [51] and have different effects on otoacoustic responses than contralateral elicitors [25, 70]. For example, the OAE phase changes by the MOCR has been shown to differ depending on the laterality of the elicitor [25, 70], and the change of OAE amplitude by the MOCR may differ depending on elicitor laterality and the elicitor bandwidth [28]. It remains unclear whether elicitor laterality influenced the results of these studies. Other potential factors include inherent variability in participants' responses and small sample sizes, with many studies including 12 or fewer subjects. High-variance data combined with low sample sizes reduces statistical power and can affect the reliability of inferential conclusions. Variability in MOCR data may stem from differences in neural responses [26, 40, 55, 56, 79, 80], or from the methodology and analyses used to measure MOCR effects [46, 56, 68].

With many of these potential factors we outlined here, in our view, Marrufo-Pérez et al. (2021) conducted the most well controlled and robust study comparing a psychoacoustic task thought to be related to MOCR gain reduction (signal in noise task) and measuring the change in TEOAE and DPOAE IO function by the elicitor. The tone detection task was signal threshold in quiet with and without a 60 dB SPL long duration broadband contralateral elicitor (850 ms duration; 0.01–10 kHz bandwidth). Pure tone frequencies were 0.500, 1.5, and 4 kHz (each 300 ms in duration). The signal started 500 ms after the elicitor onset when the elicitor was present, allowing for MOCR build to fully activate. For all signal frequencies, the addition of the elicitor increased signal threshold. TEOAE and DPOAE IO functions over a range of click levels (48–60 dB pSPL) and over a range of levels of primary tone *f*2 (30–50 dB SPL) with the level of primary tone f1 at a level ratio relative to *f*2 (*L*1 = 0.4*L2 + 39 dB SPL), respectively. They measured these functions with and without the presence of the elicitor, where the elicitor was longer than in the behavioral experiment, and was continuously presented contralaterally during the elicitor present condition for both TEOAEs and DPOAEs. TEOAE level was estimated in various frequency bands, including 1, 1.5, 2, 3, 4 kHz. Primary *f*2 frequencies were 1, 1.5, 2, 3, and 4 kHz, with a *f*2/*f*1 ratio fixed at 1.2. They found that the elicitor suppressed the OAE at all frequency bands (TEOAEs) and *f*2 frequencies (DPOAEs) tested, respectively [46].

Most OAE studies measuring MOCR effects estimate the suppression of the response, which is the decibel change or percent change of the OAE magnitude by the elicitor, and is known as the vertical displacement of the OAE on the cochlear IO function (see Supplementary File 1; Supplementary Fig. 1D; [46] their Fig. 1). Marrufo-Pérez and colleagues note that comparing the elicitor induced signal threshold increase in behavior to the negative decibel change would not be a fair comparison, because their behavioral task measures the horizontal shift (“effective attenuation”) while suppression of OAEs would measure the vertical shift of the cochlear IO function. Therefore, they quantified the horizontal displacement of the TEOAE and DPOAE IO functions by fitting straight lines to their functions, with and without the elicitor. It should be noted that differences in horizontal and vertical displacement likely occur over the compressive part of the IO function (for mid- and high probe and signal levels), but the horizontal and vertical displacement should be the same when the probe and signal levels are on the lower linear part of the IO function [46, 68].

Horizontal displacement of the TEOAE IO function was calculated by estimating the TEOAE level in the fitted line without the elicitor produced by a 54 dB pSPL click followed by the click level on the TEOAE IO function with the elicitor present that produced the same TEOAE level (often estimated by extrapolation). TEOAE IO horizontal displacement is the difference in click level between these two conditions [46, 68]. DPOAE IO horizontal displacement was calculated similarly, where the displacement was estimated relative to the DPOAE response for the *L*2 = 35 dB SPL. Both of their TEOAE and DPOAE probe levels are on the lower portion of the IO function. Correlational comparisons were made between the TEOAE and DPOAE IO curve horizontal displacements and the tone threshold shifts by the elicitor. These comparisons were made at 1.5 kHz and 4 kHz, corresponding to the TEOAE frequency band, the DPOAE for the *f*2 frequency, or the psychoacoustic signal frequency. Overall, none of their comparisons were statistically significant, and the relationship between the measures in all comparisons trended towards being flat. Their findings are in despite of each of their measures producing effects that are consistent with MOCR activation by a contralateral broadband elicitor (an increase in signal threshold and decrease in OAE magnitude), they used comparable methodology between their psychoacoustic and physiological measures, they compared the horizontal displacement for fair comparison, and they compared relatively large sample sizes (7–9 subjects at 1.5 kHz, and 13–15 subjects at 4 kHz). The results from Marrufo-Pérez et al., 2021 are nearly identical to the results of the comparisons made in the current study. This suggests that even highly controlled and robust studies, such as the Marrufo-Pérez et al., 2021 study and the current study, psychoacoustic and otoacoustic estimates of the magnitude of MOCR gain reduction are unlikely to positively correlate even though both measures activate the MOCR.

We note that individual IO functions, measured psychoacoustically and by OAEs, can vary across subjects. The linear and compressive regions, as well as the knee point separating them, are not fixed in level and may differ from one individual to another. Because we used a single probe click level, we were unable to determine whether the measurements in each subject were part of the linear or compressive portion of the IO function. It is possible this may have added to the variability found in the subject responses. Nonetheless, based on our preliminary testing of TEOAE magnitude and click-level effects, and findings from similar TEOAE paradigms, the choice of a 54 dB FPL probe level provided a reasonable trade-off between TEOAE signal-to-noise ratio and the magnitude of observable MOCR effects (Supplementary File 1; Supplementary Fig. 1). Also, as mentioned, we were unable to test for the presence of SSOAEs and did not assess the presence of SOAEs. The presence of either could contaminate the TEOAE recordings, potentially confounding the results and introducing unwarranted variability. It remains unknown whether SSOAEs or SOAEs were present in our measurements.

Cognitive and attentional differences between psychoacoustic and physiological measures also warrant consideration. Psychoacoustic tasks require active listening, with participants responding to auditory and visual cues, whereas OAE measurements typically involve passive listening, such as watching a silent program or having no visual stimulus. Selective visual attention has been shown to activate the MOCR and reduce cochlear gain [81], potentially leading to differences between psychoacoustic and OAE measures [11, 15, 46]. However, this issue remains controversial, with some studies reporting attentional effects (e.g., [82]) and others finding none (e.g., [83]).

Other studies have shown that comparing different types of physiological MOCR measurements in the same subjects often yields non-correlative outcomes. For instance, Lichtenhan et al. (2016) measured MOCR effects in the compound action potential (CAP) and the DPOAE in humans using a contralateral broadband elicitor [79]. They found that changes in the DPOAE were not predictive of changes in the CAP, with responses varying substantially among subjects—some exhibited weaker responses in one measure and stronger responses in the other [79]. Similarly, Puria et al. (1996) examined changes in the CAP and DPOAE with a contralateral broadband elicitor in lightly anesthetized cats. Their results showed large differences in the magnitude of the measures and significant variability, leading to a lack of predictive relationships between them [80]. These findings indicate that even when highly controlled measures of MOCR gain reduction are obtained in the same subjects, the magnitudes of the responses may not correlate. Additional factors contributing to the mixed results and lack of consistent relationships between these measures have been discussed elsewhere [11, 46].

In this study, considerable effort was made to optimize experimental and stimulus design to detect any potential statistical relationships between psychoacoustic and physiological measures of gain reduction. A within-subjects repeated-measures design with a relatively large sample size was used to enhance statistical power, addressing the smaller sample sizes in prior research. An LMM model accounted for individual variability in the statistical analyses. A forward masking paradigm was employed to eliminate suppression effects, and highly similar stimuli were used across measures, unlike many previous studies. Refined OAE analyses incorporated both magnitude and phase, focusing on a narrow frequency band centered at the psychoacoustic signal frequency—an approach not taken in prior studies, which typically analyzed only OAE magnitude across broader frequency ranges. Despite these methodological improvements, TEOAE responses were not reliable predictors of psychoacoustic responses when directly correlating the magnitude and time constants of gain reduction. This outcome suggests that one or more of the previously discussed factors (or others not mentioned) may have contributed to the lack of correlation in this study. This underscores the importance of using datasets from diverse approaches—psychoacoustic, physiological, neural, and modeling—to fully understand MOCR gain reduction.

### Interpreting MOCR Induced Phase Changes

The combination of TEOAE magnitude and phase in the response (ΔTEOAE_m+p_) appears to produce elicitor-induced effects that are more evident compared to changes in TEOAE magnitude alone (ΔTEOAE_m_) or phase alone (ΔTEOAE_p_) (Supplementary File 3; Supplementary Fig. 2). Both findings are consistent with a recent study measuring contralateral broadband elicitor induced MOCR effects on TEOAE IO functions using metrics consistent with those used in the current study (ΔTEOAE_m_, ΔTEOAE_m+p_, and ΔTEOAE_p_) [68]. Their ΔTEOAE_m+p_ estimate was always larger than their ΔTEOAE_m_ and ΔTEOAE_p_ conditions for all of the probe click levels tested ([68] their Fig. 5). Similar to that study, the current studies ΔTEOAE_m+p_ responses also exhibit considerably more variance than ΔTEOAE_m_ responses, as shown in Figs. 6 and 7 and the LMM output (Table 4). As mentioned previously, our ΔTEOAE_p_ data were quite variable across subjects (Supplementary File 3; Supplementary Fig. 2). The sources of this additional variance are not fully understood, but several possibilities are considered below.

The phase of reflection-source OAEs (TEOAEs and SFOAEs) depends on the frequency of the probe used to evoke the response [71]. This frequency dependency arises from the cochlear traveling wave's delay at its peak, which is inherently frequency dependent. At the wave's characteristic frequency, sound stimulation generates multiple reflection sources within the cochlea due to intrinsic irregularities or "roughness" in its structure [71]. These reflections interact and interfere, shaping the overall phase response of the OAE. Reflection-source OAEs are intrinsically variable due to the cochlea's roughness, which differs among individuals and contributes to unique OAE microstructures and phase responses. The bandwidth of the analyzed frequency region influences the observed phase response through its effect on wave-scattering interactions [84]. In this study, the TEOAE analysis used a 1/3-octave band centered at 2 kHz, encompassing many frequencies with corresponding phases that collectively contribute to the overall phase. Variability in these responses may arise from individual differences in cochlear roughness, analysis bandwidth, and experimental stimuli.

It remains unclear why incorporating phase in the TEOAEs (ΔTEOAE_m+p_) shortened the MOCR time constants, making them more similar to psychoacoustic time constants. One possibility is that OAE phase can be used to estimate the OAE group delay [85]. Reflection-source OAE group delay has been used to infer cochlear frequency tuning, with these estimates aligning closely with psychoacoustic measures of tuning, particularly when forward masking is used [86, 87]. Elicitor-induced MOCR activation may broaden cochlear filter tuning, leading to shorter (decreased) TEOAE latencies [69, 88]. Mertes and Goodman (2016) observed that elicitor-induced TEOAE effects typically result in decreased magnitude and phase leads, consistent with MOCR inhibition and reduced TEOAE latency. This may explain why ΔTEOAE_m+p_ time constants were shorter than ΔTEOAE_m_ time constants; the ΔTEOAE_m+p_ metric might account for both changes in filter shape and gain at the cochlear filter output. However, caution is warranted with this interpretation. Studies have reported that elicitor-induced OAE phase changes can result in either phase leads or lags [26, 68, 69], a trend we also observed in our individual ΔTEOAE_p_ data. The averaged ΔTEOAE_p_ response exhibited a small, non-significant trend toward increasing phase lead with elicitor duration, ranging from approximately 0.7° to 2.3° (Supplementary File 3; Supplementary Fig. 2). The relevance of OAE phase to MOCR gain reduction remains unclear [25, 26, 68–70]. Further research is needed to understand the mechanical changes causing phase shifts and whether these changes hold significance for auditory perception [70].

### Implications of the Temporal Dynamics of Gain Reduction with Broadband Elicitor Duration

MOCR-mediated gain reduction is a mechanism that can facilitate neural adaptation to sound stimulation over timescales ranging from tens of milliseconds to the hundreds of milliseconds in humans [2, 24, 32, 33]. These timescales likely play a critical role in dynamic neural encoding in environments that change rapidly in both sound intensity and frequency [89]. The time constants and buildup of gain reduction observed in our psychoacoustic and physiological data aligns with the fast time constant and buildup of the MOCR. Neural adaptation supports speech intelligibility in noise [1], and individuals with cochlear hearing impairment exhibit reduced adaptation to noise, which likely contributes to their poorer performance in speech-in-noise tasks [16]. Although the degree to which the MOCR contributes to neural adaptation in humans remains debated, evidence suggests that cochlear hearing loss leads to reduced adaptation effects to preceding sounds [20, 90, 91]. Diminished adaptation to noise because of cochlear hearing loss may also result in an altered time course of gain reduction due to weaker afferent input. Future studies using methods similar to those employed here could test this hypothesis in individuals with cochlear hearing loss by measuring time constants and the buildup of gain reduction. Psychoacoustic methods may be particularly effective at measuring this effect, as the effects we observed (4–9 dB) were substantially larger than those measured via ΔTEOAE (0.5–1.5 dB).

Forward masking paradigms were used to study changes in cochlear gain. In the psychoacoustic experiment, signal thresholds were measured near the detection level, resulting in increased signal thresholds. In the TEOAE experiment, a low-level probe was used (54 dB peFPL), and the change in the response resulted in a decrease in TEOAE level. These conditions were designed to maximize gain reduction effects while controlling for other factors, such as suppression and the MEMR. In realistic listening scenarios, such as crowded or noisy environments (e.g., a busy park or restaurant), where MOCR gain reduction is most beneficial, sounds of interest (e.g., speech or localized directional cues) and background noise overlap temporally at suprathreshold levels. In these situations, MOCR gain reduction likely continuously adapts the auditory system's dynamic range across varying sound levels, with two-tone suppression also contributing to this adjustment. Gain reduction moves the compression knee of the BM IO function to higher input levels, linearizing (increasing) its slope across a range [15, 32, 39], which may enhance sensitivity to perceptual intensity changes [10]. Gain reduction is likely fully active in such environments, as background noise is continuously present. In addition to the shorter timescales of gain reduction observed in our data and in previous studies, longer timescales of gain adjustment, on the order of tens of seconds, have also been reported. However, the origins and functional significance of these longer timescales remain less understood [24, 29–33]. One possibility is that the MOCR system continuously adjusts the auditory dynamic range on both fast and slow timescales to optimize listening conditions in response to varying environmental and acoustic conditions that are present.

### Conclusions

The effects of ipsilateral broadband elicitor duration on gain reduction were measured using forward masking psychoacoustic and TEOAE paradigms in adults with normal hearing. Gain reduction was measured by changes in signal thresholds and TEOAE levels by the elicitors, with time constants estimated from these measurements. On average, psychoacoustic and physiological time constants were similarly short (< 100 ms) and showed a buildup that was maximal or near-maximal within 200 ms of elicitor onset. These findings are consistent with cochlear gain reduction, and likely reflect the fast timescale and buildup of MOCR gain reduction. The observed timescales of gain reduction were notably shorter than those typically associated with elicitors used in previous psychoacoustic and physiological studies, which often last hundreds of milliseconds or are continuously presented. Despite both measures being consistent with cochlear gain reduction, direct comparisons of the physiological and psychoacoustic magnitude changes and time constants yielded mixed results including non-significant correlations. This lack of relationship may be influenced by factors such as individual variability, differences in methodology and analyses, and/or other factors.

## Supplementary Information

Below is the link to the electronic supplementary material.

ESM 1 (EPS 303 KB)

ESM 2 (PDF 32 KB)

ESM 3 (EPS 49, 945 KB) 

## Data Availability

The data and code that support the findings of the current study have not been made publicly available. The data and code are, however, available from the authors upon reasonable request.

## Abbreviations

CAP: Compound action potential
DPOAEs: Distortion-product otoacoustic emissions
IO function: Input-output function
FPL: Forward pressure level
LMM: Linear mixed effects model
MEMR: Middle ear muscle reflex
MOCR: Medial olivocochlear reflex
OAEs: Otoacoustic emissions
PTC: Psychoacoustic tuning curve
SFOAEs: Stimulus-frequency otoacoustic emissions
SOAEs: Spontaneous otoacoustic emissions
SSOAEs: Synchronized spontaneous otoacoustic emissions
TEOAEs: Transient-evoked otoacoustic emissions
ΔTEOAE_m_: Change in the TEOAE magnitude by the elicitor
ΔTEOAE_m__+p_: Change in the TEOAE magnitude and phase by the elicitor, calculated by vector subtraction
ΔTEOAE_p_: Change in phase angle (degrees) of the TEOAE by the elicitor
Δt^PSY^ on/on: Total duration from the onset of the elicitor to the onset of the signal
Δt^OAE^ on/on: Total duration from the onset of the elicitor to the onset of the click
Δt^PSY^ on/on - 20 ms: Total duration of the elicitor to the onset of the signal while accounting for the onset delay of the MOCR (20 ms) for psychoacoustic time constant estimation
Δt^OAE^ on/on - 20 ms: Total duration of the elicitor to the onset of the click while accounting for the onset delay of the MOCR (20 ms) for TEOAE time constant estimation
WAI: Wideband acoustic immittance
