# Supplementary material for: The Effects of Broadband Elicitor Duration on Transient-Evoked Otoacoustic Emissions and a Psychoacoustic Measure of Gain Reduction
Source: J Assoc Res Otolaryngol. 2025 Nov 25;26(6):671–97. doi: 10.1007/s10162-025-01011-1 (PMC12698501; doi:10.1007/s10162-025-01011-1)
Supplement: Supplementary file 2 — (PDF 32 KB) [file 10162_2025_1011_MOESM2_ESM.pdf]

Subjects	Quiet thresholds	On-frequency masker thresholds	Off-frequency masker thresholds	On-frequency signal thresholds	Off-frequency signal thresholds	Gain
S1	26.5	27.0	71.0	29.5	30.4	44.0
S2	23.5	24.0	74.0	30.6	30.8	50.0
S3	23.6	21.0	68.0	31.0	31.8	47.0
S4	24.5	25.6	67.0	30.6	30.8	41.4
S5	23.3	22.0	63.0	28.9	29.6	41.0
S6	29.5	26.0	64.0	36.9	35.6	38.0
S7	29.5	25.0	71.0	35.0	35.6	46.0
S8	20.9	18.2	60.2	26.0	24.8	42.0
S9	22.9	19.7	57.0	27.6	28.6	37.3
S10	20.6	17.3	72.0	24.9	23.8	54.7
S11	22.4	19.3	64.4	28.8	28.1	45.1
S12	26.3	24.4	64.5	32.1	29.1	40.1
S13	25.9	24.5	62.4	29.8	30.0	37.9
S14	26.6	23.3	66.2	29.8	29.8	42.9
S15	26.8	21.6	62.8	32.8	33.6	41.2
S16	20.8	17.8	57.0	26.5	25.8	39.2
S17	27.9	27.6	59.4	31.6	32.0	31.8
S18	27.6	22.8	57.9	29.0	29.3	35.1
S19	30.5	28.4	55.0	35.0	36.8	26.6
Average	25.2	22.9	64.0	30.3	30.3	41.1
